# Does Consumption of Ultra-Processed Foods Matter for Liver Health? Prospective Analysis among Older Adults with Metabolic Syndrome

**DOI:** 10.3390/nu14194142

**Published:** 2022-10-05

**Authors:** Jadwiga Konieczna, Miguel Fiol, Antoni Colom, Miguel Ángel Martínez-González, Jordi Salas-Salvadó, Dolores Corella, María Trinidad Soria-Florido, J. Alfredo Martínez, Ángel M. Alonso-Gómez, Julia Wärnberg, Jesús Vioque, José López-Miranda, Ramon Estruch, M. Rosa Bernal-López, José Lapetra, Lluís Serra-Majem, Aurora Bueno-Cavanillas, Josep A. Tur, Vicente Martín Sánchez, Xavier Pintó, José J. Gaforio, Pilar Matía-Martín, Josep Vidal, Clotilde Vázquez, Lidia Daimiel, Emilio Ros, Maira Bes-Rastrollo, María Pascual, Jose V. Sorlí, Albert Goday, María Ángeles Zulet, Anai Moreno-Rodriguez, Francisco Jesús Carmona González, Rafael Valls-Enguix, Juana M. Janer, Antonio Garcia-Rios, Rosa Casas, Ana M. Gomez-Perez, José Manuel Santos-Lozano, F. Javier Basterra-Gortari, María Ángeles Martínez, Carolina Ortega-Azorin, Joan Bayó, Itziar Abete, Itziar Salaverria-Lete, Miguel Ruiz-Canela, Nancy Babio, Lourdes Carres, Dora Romaguera

**Affiliations:** 1 Research Group on Nutritional Epidemiology & Cardiovascular Physiopathology (NUTRECOR), Health Research Institute of the Balearic Islands (IdISBa), University Hospital Son Espases (HUSE), 07120 Palma de Mallorca, Spain; 2 Centro de Investigación Biomédica en Red Fisiopatología de la Obesidad y la Nutrición (CIBEROBN), Institute of Health Carlos III, 28029 Madrid, Spain; 3 Department of Preventive Medicine and Public Health, IDISNA, University of Navarra, 31008 Pamplona, Spain; 4 Department of Nutrition, Harvard T.H. Chan School of Public Health, Boston, MA 02115, USA; 5 Universitat Rovira i Virgili, Departament de Bioquímica i Biotecnologia, Unitat de Nutrició, 43201 Reus, Spain; 6 University Hospital of Sant Joan de Reus, Nutrition Unit, 43204 Reus, Spain; 7 Institut d’Investigació Sanitària Pere Virgili (IISPV), 43204 Reus, Spain; 8 Department of Preventive Medicine, University of Valencia,46010 Valencia, Spain; 9 Unit of Cardiovascular Risk and Nutrition, Institut Hospital del Mar de Investigaciones Médicas Municipal d’Investigació Médica (IMIM), 08003 Barcelona, Spain; 10 Department of Nutrition, Food Sciences, and Physiology, University of Navarra, IDISNA, 31008 Pamplona, Spain; 11 Precision Nutrition and Cardiometabolic Health Program, IMDEA Food, CEI UAM + CSIC, 28049 Madrid, Spain; 12 Bioaraba Health Research Institute, Cardiovascular, Respiratory and Metabolic Area, Osakidetza Basque Health Service, Araba University Hospital, University of the Basque Country UPV/EHU, 01009 Vitoria-Gasteiz, Spain; 13 Department of Nursing, University of Málaga, Institute of Biomedical Research in Malaga (IBIMA), 29016 Málaga, Spain; 14 CIBER de Epidemiología y Salud Pública (CIBERESP), Instituto de Salud Carlos III, 28029 Madrid, Spain; 15 Alicante Institute for Health and Biomedical Research (ISABIAL-UMH), 03010 Alicante, Spain; 16 Department of Internal Medicine, Maimonides Biomedical Research Institute of Cordoba (IMIBIC), Reina Sofia University Hospital, University of Cordoba, 14004 Cordoba, Spain; 17 Department of Internal Medicine, Institut d’Investigacions Biomèdiques August Pi Sunyer (IDIBAPS), Hospital Clinic, University of Barcelona, 08007 Barcelona, Spain; 18 Department of Internal Medicine, Regional University Hospital of Malaga, Instituto de Investigación Biomédica de Málaga (IBIMA), University of Málaga, 29009 Málaga, Spain; 19 Department of Family Medicine, Research Unit, Distrito Sanitario Atención Primaria Sevilla, 41013 Sevilla, Spain; 20 Research Institute of Biomedical and Health Sciences (IUIBS), University of Las Palmas de Gran Canaria & Centro Hospitalario Universitario Insular Materno Infantil (CHUIMI), Canarian Health Service, 35001 Las Palmas de Gran Canaria, Spain; 21 Department of Preventive Medicine and Public Health, University of Granada, 18011 Granada, Spain; 22 Research Group on Community Nutrition & Oxidative Stress, University of Balearic Islands, 07122 Palma de Mallorca, Spain; 23 Institute of Biomedicine (IBIOMED), University of León, 24071 León, Spain; 24 Lipids and Vascular Risk Unit, Internal Medicine, Hospital Universitario de Bellvitge Idibell-UB, Hospitalet de Llobregat, 08907 Barcelona, Spain; 25 Departamento de Ciencias de la Salud, Centro de Estudios Avanzados en Olivar y Aceites de Oliva, Universidad de Jaén, 23071 Jaén, Spain; 26 Department of Endocrinology and Nutrition, Instituto de Investigación Sanitaria Hospital Clínico San Carlos (IdISSC), 28040 Madrid, Spain; 27 CIBER Diabetes y Enfermedades Metabólicas (CIBERDEM), Instituto de Salud Carlos III (ISCIII), 28029 Madrid, Spain; 28 Department of Endocrinology, Institut d’ Investigacions Biomédiques August Pi Sunyer (IDIBAPS), Hospital Clinic, University of Barcelona, 08007 Barcelona, Spain; 29 Department of Endocrinology and Nutrition, Hospital Fundación Jimenez Díaz, Instituto de Investigaciones Biomédicas IISFJD, University Autonoma, 28040 Madrid, Spain; 30 Nutritional Control of the Epigenome Group, Precision Nutrition and Obesity Program, IMDEA Food, CEI UAM + CSIC, 28049 Madrid, Spain; 31 Lipid Clinic, Department of Endocrinology and Nutrition, IDIBAPS, Hospital Clínic, 08036 Barcelona, Spain; 32 Departament de Medicina, Universitat Autònoma de Barcelona, 08193 Barcelona, Spain; 33 Unidad de Gestión Clínica Torrequebrada, Distrito de Atención Primaria Costa del Sol, Servicio Andaluz de Salud, 29640 Benalmádena, Spain; 34 Raval Health Care Centre, 03203 Elche, Spain; 35 Centro de Salud Camp Redó (UBS Son Sardina) Gerència Atenció Primària de Mallorca, 07010 Palma de Mallorca, Spain; 36 Virgen de la Victoria Hospital, Department of Endocrinology, Instituto de Investigación Biomédica de Málaga (IBIMA), University of Málaga, 29071 Málaga, Spain; 37 Servicio de Endocrinología. Complejo Hospitalario de Navarra, Servicio Navarro de Salud, 31003 Pamplona, Spain; 38 CAP El Clot, Institut Català de la Salut, 08018 Barcelona, Spain; 39 Atención Primaria Sant Martí, Institut Català de la Salut, 08020 Barcelona, Spain

**Keywords:** ultra-processed foods, liver health markers, fatty liver index, hepatic steatosis index, metabolic syndrome

## Abstract

Non-alcoholic fatty liver disease (NAFLD) includes a spectrum of liver alterations that can result in severe disease and even death. Consumption of ultra-processed foods (UPF) has been associated with obesity and related comorbidities. However, the link between UPF and NAFLD has not been sufficiently assessed. We aimed to investigate the prospective association between UPF consumption and liver health biomarkers. **Methods:** We followed for 1 year 5867 older participants with overweight/obesity and metabolic syndrome (MetS) from the PREDIMED-Plus trial. A validated 143-item semi-quantitative food frequency questionnaire was used to evaluate consumption of UPF at baseline, 6, and 12 months. The degree of processing for foods and beverages (g/day) was established according to the NOVA classification system. The non-invasive fatty liver index (FLI) and hepatic steatosis index (HSI) were used to evaluate liver health at three points in time. The associations between changes in UPF consumption (percentage of total daily dietary intake (g)) and liver biomarkers were assessed using mixed-effects linear models with repeated measurements. **Results:** In this cohort, UPF consumption at baseline was 8.19% (SD 6.95%) of total daily dietary intake in grams. In multivariable models, each 10% daily increment in UPF consumption in 1 year was associated with significantly greater FLI (β 1.60 points, 95% CI 1.24;1.96 points) and HSI (0.43, 0.29; 0.57) scores (all *p*-values < 0.001). These associations persisted statistically significant after adjusting for potential dietary confounders and NAFLD risk factors. **Conclusions:** A higher UPF consumption was associated with higher levels of NAFLD-related biomarkers in older adults with overweight/obesity and MetS.

## 1. Background

Excessive liver fat accumulation in the absence of alcoholism, known as non-alcoholic fatty liver disease (NAFLD), includes a spectrum of fat-liver alterations affecting approximately 30% of adults worldwide, but its prevalence is increased in persons with type 2 diabetes (70%) and morbid obesity (90%) [1,2]. Although it remains asymptomatic during long time, excess liver fat is an important cause of morbimortality from cardiovascular disease (CVD) and malignancy, in addition to chronic liver disease. Unfortunately, at present, there are gaps in NAFLD diagnosis and limited treatment options [1,2].

Along with physical activity (PA), dietary modifications are considered the cornerstone for NAFLD management [1]. A growing body of evidence suggests that dietary fiber intake and the Mediterranean diet (MedDiet) [3] might be protective, whereas saturated and trans fatty acids (FA), simple sugars, red and processed meat, sugar-sweetened beverages (SSB), and the Western dietary pattern might act as risk factors [4]. However, with the exception of small trials assessing interventions with the MedDiet [3], most of these findings were based on cross-sectional or case-control study designs.

Ultra-processed foods (UPF) are industrial formulations manufactured using a series of processes with no domestic equivalents (i.e., soft drinks, packed snacks, processed meats, and pre-prepared dishes) [5,6]. In addition to their usual poor nutritional composition (i.e., excessive calories, simple sugars, salt, poor quality fat, as well as fiber and vitamin deprivation) [5], UPF typically contains cosmetic additives and substances formed because of extensive processing during manufacturing [7]. On the other hand, UPF are highly palatable, appealing, convenient, microbiologically pure, inexpensive, accessible, and aggressively advertised [5,8]. All these factors explain the steadily increase in UPF consumption despite the health risks associated with their regular intake [9,10].

A recent study conducted in the PREvención con DIeta MEDiterránea Plus (PREDIMED-Plus) cohort found that consumption of UPF, classified according to the NOVA system [11], was associated with greater visceral and total fat accumulation [12]. Other prospective studies with adult cohorts found associations between UPF consumption and higher risks of obesity [13,14,15], CVD [16], type 2 diabetes [17,18], renal dysfunction [19], cancer [20], biological aging [21], and all-cause mortality [22]. However, the link between UPF and NAFLD has not been sufficiently assessed [23]. Only very recently a prospective study by Zhang et al. has been published, reporting a positive link between UPF and NAFLD diagnosed using ultrasonography, within the Tianjin Chronic Low-grade Systemic Inflammation and Health (TCLSIH) Cohort Study [24] after a median of 4.2 years of follow-up; but they only measured UPF at baseline, without repeating the dietary assessment during follow-up. Therefore, our aim was to prospectively investigate how concurrent changes in consumption of UPF were associated with liver health in older individuals with overweight/obesity and the metabolic syndrome (MetS) from the Mediterranean area, using three repeated measurements of diet and biomarkers related to NAFLD throughout a 1-year follow-up of PREDIMED-Plus trial.

## 2. Methods

### 2.1. Study Overview and Population

This study corresponds to longitudinal analyses nested in the ongoing PREDIMED-Plus trial, with data collected at baseline and during the first year of follow-up. Details about the study protocol have been described elsewhere [25,26], and are available at www.predimedplus.com. Briefly, PREDIMED-Plus is a 6-year parallel-group, multicenter randomized clinical trial, aimed to assess the effectiveness of a lifestyle intervention—energy-restricted (er) MedDiet, PA promotion, and behavioral support—on the primary prevention of CVD and weight loss in older individuals with overweight/obesity harboring MetS. The control group received usual health care and recommendations to follow the MedDiet, without advice on energy restriction or PA promotion. The trial was launched in 2013 (the recruitment finished at the end of 2016) in 23 centers across Spain. Community-dwelling older men and women (55–75 years), with body mass index (BMI) ≥ 27 and <40 kg/m^2^), fulfilling ≥ three criteria for the MetS [27], but free of CVD at baseline, were invited to participate in the trial. Exclusion criteria included self-declared liver disease (chronic hepatitis or cirrhosis), therapy with immunosuppressive drugs, cytotoxic agents or systemic corticosteroids, alcohol abuse or addiction (defined as total daily alcohol intake > 50 g within past 6 months), history of inflammatory bowel disease, and active malignant cancer or history of malignancy within the last 5 years. For a small subset of participants sharing the same household, the randomization was performed as clusters (the couple was used as the unit of randomization). All participants provided written informed consent to a protocol approved by the Research Ethic Committees of all recruiting centers according to the ethical standards of the Declaration of Helsinki. The trial was registered at http://www.isrctn.com/ (ISRCTN89898870).

Out of the 6874 participants randomized for the PREDIMED-Plus trial, participants with history of liver cancer within >5 years before inclusion (*n* = 2), with missing data on variables of interest at baseline (*n* = 811), and those who were outside predefined limits for total daily energy intake (<500 or >3500 kcal for women, <800 or >4000 kcal for men) [28] at baseline and during follow-up (*n* = 194) were excluded from the analysis. After exclusions, a total of 5867 participants were included in the final analyses (see the flowchart of study participants in online Supplementary Figure S1).

### 2.2. Dietary Habits and Nutrient Intake Assessments

Dietary intake was assessed by trained dietitians during face-to-face interviews at baseline, 6-, and 12-month follow-up visits, using a semi-quantitative food frequency questionnaire (FFQ), repeatedly validated for the Spanish population [29,30]. Intakes of 143 foods and beverages (except water) were calculated by multiplying the common portion size by average consumption frequency (9 possible responses, from never to >6 times/day) over the last year (at baseline visit and over 6-month period at each follow-up visit). Daily intake of beverages was collected in cubic centimeters and then converted into milliliters (1 cc = 1 mL), and further into grams, assuming that 1 mL = 1 g. Food composition tables developed specifically for Spain [31] were used to derive nutrient (sodium(native and added in the form of salt), and cholesterol (both in mg/day), saturated and trans FA, fiber, and alcohol (all in g/day)), as well as total energy intake (kcal/day). The glycemic load was also calculated for each item taking into account the quality (glycemic index) and the amount of carbohydrate as previously described [32]. The glycemic index was determined using average values from the International Tables [33].

### 2.3. Dietary Data Processing-Based Classification and Ultra-Processed Foods

Items in the FFQ were classified according to the NOVA system [5,11] developed by the Public Health Faculty of the University of São Paulo in Brazil. This system classifies foods and beverages according to the nature, extent, and purpose of their industrial processing into four groups: (1) unprocessed or minimally processed foods (i.e., fresh or frozen fruits and vegetables, eggs, pasteurized milk, meat, seeds, nuts, grains, or plain yogurt); (2) processed culinary ingredients (i.e., oils, fats, sugar, and salt); (3) processed foods (i.e., canned vegetables, canned fish, fruits in syrup, cheeses, fresh bread, beer, and wine); and (4) UPF (i.e., soft drinks, sweet, or savory packed snacks, processed meats, pre-prepared frozen dishes, and ‘instant’ products). Details about the allocation of FFQ items to processing groups with examples are provided in online Supplementary Table S1. Furthermore, items belonging to the UPF group (foods and beverages) were allocated into the following subgroups: dairy products; processed meat; pre-prepared dishes, snacks and fast-foods; sweets; non-alcoholic beverages; and alcoholic beverages (Table 1).

Repeated data on the percentage of UPF consumption (and other NOVA groups) was computed as the sum of grams per day consumed from items in the UPF group (determined at baseline, 6-, and 12-month follow-up visits), divided by the total grams of all items consumed per day, and multiplied by 100.

### 2.4. Socio-Demographic, Lifestyle, Anthropometric, and Clinical Variables Assessment

Information on socio-demographics and health-related issues was collected from participants using a general questionnaire at baseline. Educational level, indicated by the highest educational qualification (professional or academic) achieved, was categorized into three groups (higher education or technician, secondary education, or primary education or less), smoking habits into four groups (current, never, ex-smoker, or insufficient data), whereas history of overweight was categorized into five groups (since childhood, adolescence, adulthood, after childbirth, or since menopause), and prevalence of type 2 diabetes was dichotomized (yes or no).

At baseline, 6-, and 12-month follow-up visits, total leisure-time PA (METs min/week) was assessed using the validated Minnesota-REGICOR short physical activity questionnaire [34] and sedentary behavior (SB) (h/day) by the validated Spanish version of the Nurses’ Health Study questionnaire [35]. Adherence to the erMedDiet was determined using a 17-item screener, a modified version of a validated 14-item questionnaire [36].

At each visit, trained staff measured in duplicate height and weight according to the study´s protocol using a wall-mounted stadiometer and calibrated scales, respectively. Blood pressure was measured in triplicate using a calibrated oscillometer. Averages of these repeated measurements were taken for analyses. BMI was calculated by dividing weight (kg) by squared height (m), and waist circumference (cm) was determined midway between the lowest rib and the iliac crest using an anthropometric tape.

Fasting blood samples were collected at baseline and thereafter to quantify levels of alanine aminotransferase (ALT, U/L), aspartate aminotransferase (AST, U/L), gamma-glutamyl transferase (GGT, U/L), glucose (mg/dL), high-density lipoprotein cholesterol (HDL-c, mg/dL), triglyceride (mg/dL), and glycated hemoglobin (HbA1c, %) using standard methods. MetS components were ascertained according to guidelines from the International Diabetes Federation/National Heart, Lung and Blood Institute/American Heart Association (2009), namely: waist circumference ≥ 102 cm for men and ≥88 cm for women, triglycerides ≥ 150 mg/dL, HDL-c < 40 mg/dL in men and <50 mg/dL in women, systolic blood pressure ≥ 130 and/or diastolic blood pressure ≥ 85 mmHg (or antihypertensive drug treatment), and fasting glucose ≥ 100 mg/dL (or antidiabetic drug treatment) [27].

### 2.5. Outcome Assessment: Liver Health

Repeated data of the non-invasive liver health biomarkers were computed using anthropometric and biochemical data collected at baseline, 6-, and 12-month follow-up visits. The fatty liver index (FLI) and hepatic steatosis index (HSI) were used as surrogate measures of NAFLD [37,38]. FLI [37] and HSI [38] are diagnostic algorithms built upon a cluster of liver health biomarkers, including BMI, waist circumference, blood levels of triglycerides, the liver enzyme GGT, the AST/ALT ratio, type 2 diabetes status, and sex (see below). These scores have been previously validated in large populations against imaging techniques, showing high specificity and sensitivity in predicting excess liver fat, with values < 30 ruling out and values ≥ 60 (for FLI) or ≥36 (for HSI) confirming NAFLD [37,38].

FLI = (e^(0.953×ln(triglyceride)+0.139×BMI+0.718×ln(GGT)+0.053×waist circumference−15.745)^)/(1 + e^(0.953×ln(triglyceride)+0.139×BMI+0.718×ln(GGT)+0.053×waist circumference−15.745)^) × 100

HSI = 8 * ALT/AST + BMI + (2 if type 2 diabetes, 0 otherwise) + (2 if women, 0 otherwise)

### 2.6. Statistical Analyses

All analyses were performed using the entire analytical sample as an observational cohort (both study arms combined). For descriptive analyses of a participant´s characteristics means and standard deviations (SD), and numbers and percentages (%), were calculated for continuous and categorical variables, respectively. Statistical differences in baseline characteristics by baseline sex-specific quintiles of UPF consumption were assessed using one-way ANOVA or χ^2^ test, wherever appropriate. The differences in these characteristics over follow-up time were assessed using linear mixed-effects models with random intercepts at recruiting center, cluster family, and patient level.

The same approach with linear mixed-effects modelling with random intercepts (recruiting center, cluster family, and patient) was used to evaluate associations between concurrent 6-month changes in UPF consumption with changes in indices of NAFLD over the first year of follow-up. Our exposure was modelled as repeatedly measured continuous variable (per 10% increment) and as sex-specific quintiles, with the first quintile set as the reference category. The *p* for linear trend across increasing quintiles was calculated with the use of the median value for each category. In the main analyses, two sets of covariates were used. Model 1 was minimally adjusted for age at inclusion, sex, study arm, and follow-up time (months). Model 2 was additionally adjusted for baseline educational level, smoking habits (all categorical, height, as well as repeatedly measured at baseline and every 6 months thereafter PA, SB, and alcohol intake (all continuous). Selection of covariates was performed using a causal directed acyclic graph approach implemented in the DAGitty free web application [39].

Several sensitivity analyses were performed based on model 2. The potential influence of nutritional factors (characteristics of UPF) was addressed by additional adjustment for repeatedly measured intake of total energy, saturated and trans FA, cholesterol, fiber, glycemic load, sodium (individually and including all factors simultaneously in the model), as well as adherence to erMedDiet (all continuous). Moreover, we controlled for several NAFLD-related risk factors by additional adjustment for repeatedly measured BMI, waist circumference, HbA1c, and number of MetS factors (continuous), as well as for history of overweight self-reported at baseline and type 2 diabetes prevalence at baseline (both categorical). Finally, models were rerun eliminating outliers (1st and 99th percentile) from FLI and HSI, and imputing missing follow-up data (UPF, NAFLD indices and covariables) using the last observation carried forward (LOCF) method.

Additionally, the proportion mediated by nutritional factors (characteristics of UPF, and adherence to erMedDiet, as an indicator of a healthy dietary pattern) and NAFLD-related biomarkers (known risk factors and components of FLI and HSI scores) in the studied association were quantified. For this, standard steps proposed by Baron and Kenny (1986) with adjustments introduced by Iacobucci et al. [40] were followed using multicovariate-adjusted linear mixed-effects models (Model 2). Details about the method and analyses are presented in online Supplementary Text S1.

Subgroup analyses were also performed by rerunning the model for different strata at baseline: sex (men or women), age (<65 or ≥65 y), type 2 diabetes status (non-diabetics or diabetics), alcohol intake (<20/30 g/day for men/women or ≥20/30 g/day for men/women, respectively), and adherence to erMedDiet (<8 or ≥8 points). Median values were used as a threshold to stratify age and erMedDiet, whereas safe limits accepted by guidelines of scientific associations were used for alcohol intake [41]. The *p* values for interaction were computed for each scenario rerunning model 2 with a multiplicative interaction term inserted between these variables and exposure in continuous form.

In secondary analyses, model 2 was rerun to explore the associations between concurrent changes in consumption of different food subgroups within UPF (continuous variable) and NAFLD indices.

Analyses were performed using Stata v15. and statistical significances was set at *p*  <  0.05. The last actualized version of the PREDIMED-Plus longitudinal database generated on 22nd December 2020 (202012220958_PREDIMEDplus 2020) was used.

## 3. Results

Table 2 presents baseline characteristics of study participants according to quintiles of UPF consumption. The analytical sample (*n* = 5867) comprised 47.8% women with average age at inclusion of 65.0 years (SD 4.9 years). Overall obesity and abdominal obesity (73.1 and 93.0%, respectively), as well as NAFLD screened using FLI and HSI (84.1 and 95.2%, respectively), were highly prevalent. Mean UPF consumption accounted for 8.19% (SD 6.95%) of total daily intake (in grams). Compared to participants with the lowest UPF consumption (Q1, mean consumption 2.12% (SD 0.81%) of total daily intake (in grams)), participants in the highest quintile (Q5, mean UPF consumption 19.0% (SD 7.9%)) were younger and showed less healthy lifestyle habits in terms of physical inactivity; higher sedentariness; intake of energy, saturated and trans FA, cholesterol, sodium, and glycemic load; as well as lower intake of fiber, alcohol, and adherence to MedDiet (*p* < 0.001 for all comparisons). Moreover, participants in the highest quintile of UPF consumption presented higher values of BMI and WC than their counterparts in the lowest quintile, as well as higher levels of liver health biomarkers, such as blood ALT/AST ratio and triglycerides, as well as both NAFLD indices (*p* ≤ 0.001). Sweets (28%), non-alcoholic beverages (26%), and processed meats (22%) were the main food subgroups consumed within the UPF category at baseline (Table 1).

As shown in online Supplementary Table S2, lifestyle factors and liver health markers improved over time compared to the baseline (*p* < 0.05 for all comparisons). An increase in the consumption of unprocessed or minimally processed foods and decrease in products with higher degree of processing was observed during the 1-year follow-up period, probably as consequence of MedDiet recommendations, which were given to participants in both study arms (*p* < 0.001).

**Figure 1:**
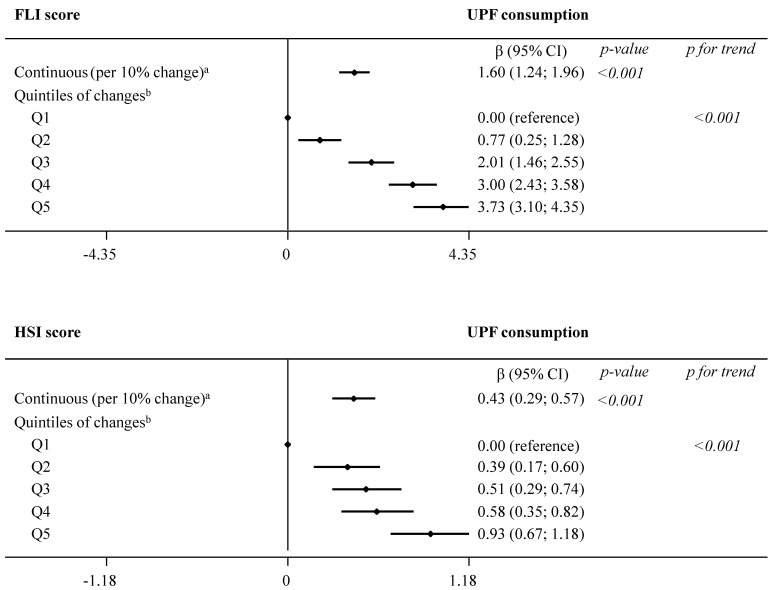
Dose–response relationship in the association between concurrent changes in UPF consumption (% of g/day) and changes in NAFLD indices during 1 year of follow-up (fully adjusted model 2). The consumption of UPF was expressed as a percentage of total food and beverage intake in g/day. Daily intake of beverages was collected in cubic centimeters and then converted into milliliters (1 cc = 1 mL), and further into grams, assuming that 1 mL = 1 g. Mixed-effects linear modelling for repeated measures with random intercepts at recruiting center, cluster family, and patient level were used after controlling in fully adjusted model 2 for baseline variables, such as age, sex, study arm, educational level, smoking habits, and height, as well as repeatedly measured physical activity, sedentary behavior, alcohol intake, and follow-up time. ^a^ Estimates β are interpreted as changes in NAFLD associated with increments of 10% in UPF consumption. ^b^ Estimates β are interpreted as changes in NAFLD indices in each sex-specific quintile of UPF consumption, compared to quintile 1, the reference category. Abbreviations: FLI—fatty liver index; HSI—hepatic steatosis index; NAFLD—non-alcoholic fatty liver disease; UPF—ultra-processed foods.

Results from the main analysis evaluating the association between concurrent changes in UPF consumption and changes in indices of NAFLD are summarized in Figure 1 and presented in detail in online Supplementary Table S3. In model 2, we observed significant (*p* < 0.001) associations between each daily 10% increment in UPF consumption and greater FLI (β 1.60, 95% CI 1.24; 1.96) and HSI (0.43, 0.29; 0.57) over the 1-year follow-up. Comparison across increasing quintiles of UPF consumption revealed a significant dose–response relationship with both NAFLD indices (*p for trend* *< 0.001*): FLI (β estimates for Q5 3.73, 95% CI 3.10; 4.35) and HSI (0.93, 0.67; 1.18).

**Figure 2:**
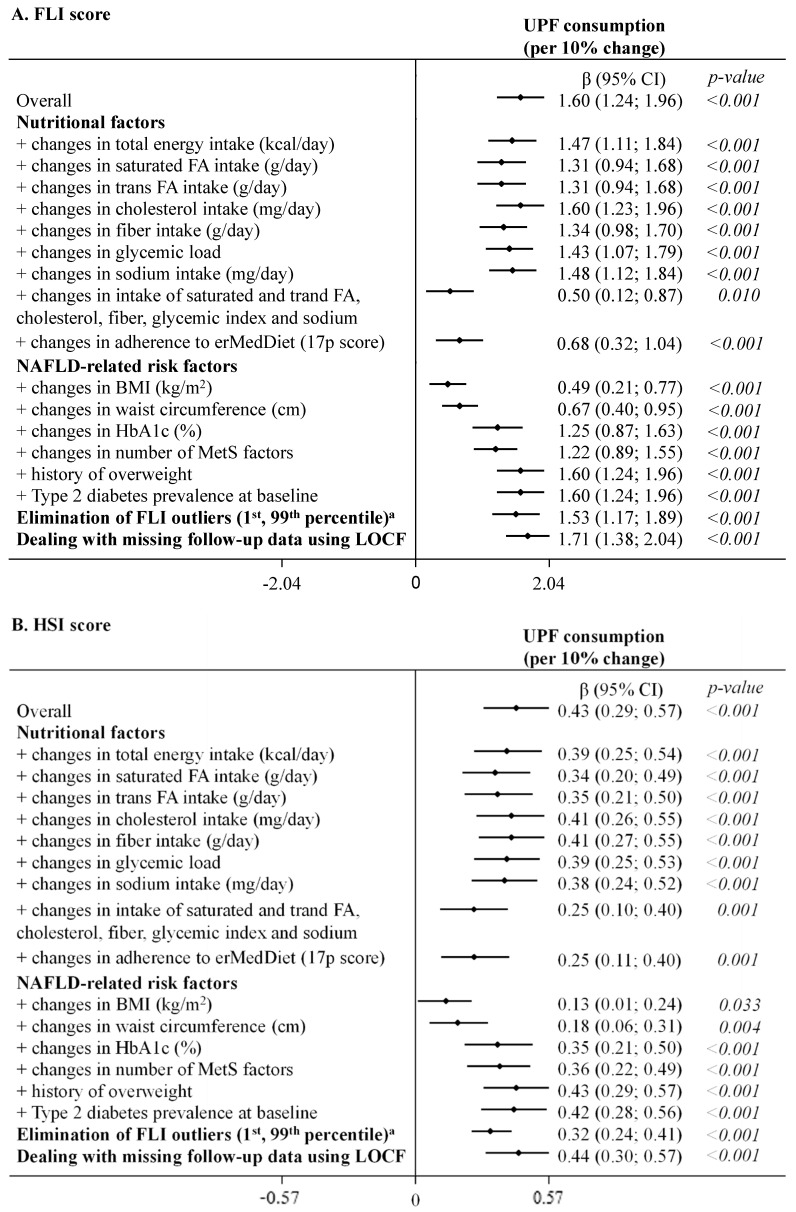
Summary of the sensitivity analysis for the association between concurrent changes in UPF consumption (% of g/day, continuous variable) and changes in NAFLD indices during 1 year of follow-up (fully adjusted model 2). The consumption of UPF was expressed as a percentage of total food and beverage intake in g/day. Daily intake of beverages was collected in cubic centimeters and then converted into milliliters (1 cc = 1 mL), and further into grams, assuming that 1 mL = 1 g. Mixed-effects linear modelling for repeated measures with random intercepts at recruiting center, cluster family and patient level were used after controlling in fully adjusted model 2 for baseline variables, such as age, sex, study arm, educational level, smoking habits, and height, as well as repeatedly measured physical activity, sedentary behavior, alcohol intake, follow-up time, and use of antidiabetic medications (for models with HbA1c). Estimates β are interpreted as changes in NAFLD indices associated with increments of 10% in UPF consumption. ^a^ Outliers (1st, 99th percentile) in the outcome variables were eliminated at baseline and follow-up (for FLI total *n* = 318, for HSI total *n* = 316). Abbreviations: BMI—body mass index; erMedDiet—energy-restricted Mediterranean Diet; FA—fatty acids; FLI—Fatty liver index; HbA1c—glycated hemoglobin; HSI—Hepatic steatosis index; LOCF—last observation carried forward; MetS—metabolic syndrome; NAFLD—non-alcoholic fatty liver disease; UPF—ultra-processed foods.

As highlighted in Figure 2 (UPF coded as continuous variable) and shown in detail in online Supplementary Table S4 (UPF coded as continuous and sex-specific quintiles), results remained statistically significant after further adjustments in sensitivity analysis. Only simultaneous adjustment for several factors related to nutritional quality of the diet (saturated and trans FA, cholesterol, fiber, glycemic load and sodium) and an adherence to erMedDiet, as well as BMI and waist circumference, decreased point estimates, yet the associations of UPF consumption with both NAFLD indices remained statistically significant. A similar pattern was observed when the exposure was coded in quintiles (online Supplementary Table S4).

In the mediation analysis, we found that changes in nutritional factors partly mediated the association with concurrent changes in NAFLD indices (Table 3 and online Supplementary Table S5). Among them, changes in nutritional characteristics of UPF, such as saturated and trans FA, mediated 17–21% of the associations for both indices, and fiber and glycemic load explained 15% and 11% of the association for FLI, respectively; whereas changes in intake of total energy, sodium, and cholesterol did not mediate any of the associations. Moreover, changes in adherence to erMedDiet acted as mediator in 58% and 43% for FLI and HSI, respectively. As far as NAFLD-related biomarkers (known risk factors and components of both scores) are concerned, changes in BMI were responsible in 69% for the association between concurrent changes in UPF consumption and both FLI and HSI; whereas, waist circumference was responsible in 56% of the association for FLI and in 82% for HSI. Furthermore, in the case of FLI, the association with UPF was driven by changes in triglycerides and MetS factors (both in 26%), followed by changes in Hba1c (14%). In turn, in case of HSI, the association was driven by the ALT/AST ratio (39%), ALT and MetS factors (both in 16%), and changes in HbA1c (15%). Changes in GGT and AST did not mediate the respective associations for FLI and HSI.

In subgroup analyses (online Supplementary Table S6), we found that the direct association between UPF consumption and FLI was slightly more pronounced in non-diabetics (β 1.73, 95% CI 1.29; 2.18, *p* < 0.001) than in diabetics (1.29, 0.68; 1.90, <0.001) (*p for interaction = 0.027*).

In additional analyses (online Supplementary Table S7), we found that all UPF subgroups contributed to observed associations with NAFLD indices. In particular, pre-prepared dishes, snacks, and fast-foods, as well as processed meats and sweets, showed the strongest statistically significant associations (all *p*-values < 0.001) with both liver scores. In turn, the subgroup of alcoholic beverages was only strongly associated with FLI.

## 4. Discussion

In this large prospective cohort study of older adults with overweight/obesity and MetS from Spain, a Mediterranean country, we found that UPF consumption was associated with worse liver health, assessed using biomarkers related to NAFLD. This direct association was ascertained using sophisticated analyses and validated tools, and was robust after accounting for a wide range of indicators related to nutritional quantity and quality of the diet and NAFLD risk.

Given the mounting body of evidence showing associations of UPF consumption with well-known risk factors for NAFLD, such as obesity [12,13,14,15], type 2 diabetes [17], or hypertension [42,43], our findings were not unexpected. Moreover, they are in line with recent evidence on the role of diet in NAFLD [4], pointing to several UPF, such as processed meat [44] and SSB [45], as culprits in objectively-determined NAFLD development. However, studies on diet-NAFLD risk have rarely been prospective, and few of them aggregated foods according to the nature, extent, and purpose of processing [23,24]. In this sense, our findings corroborate recent results from TCLSIH prospective cohort in China, showing the link between UPF consumption and risk of developing NAFLD, diagnosed using ultrasonography [24]. Here, we support this link using repeatedly measured dietary habits in a different population from a Mediterranean country. It needs to be underlined that processing not always has to be negative, as it also can increase the safety and shelf-life of foods and beverages. However, ultra-processing combines several ingredients with little, if any, intact whole foods, which results in the creation of new products with nutritionally imbalanced properties [5,6].

Several putative mechanisms of action could be responsible for the link between UPF and liver fat accumulation. The first is the nutritional characteristics of UPF, which is poor due to the industrial manipulations that they undergo. For instance, the incorporation of sizable amounts of saturated and trans FA may increase product stability and palatability [46], and these nutrients have been associated with increased liver fat in humans and rodents [4,47]. It should be mentioned that there has been significant progress towards 2023, the target for elimination of industrially produced trans FA around the world [48]. In the European Union countries, the regulation limiting the use of artificial trans FA came into force just recently (April 2021) [49]. However, given that dietary data used in this study were collected before that (2013–2017), we could still estimate the intake of trans FA in this population. Furthermore, low fiber content is a common attribute of UPF, and the breakdown of natural food matrix during ultra-transformation might also reduce its quantity. Recent findings from a large cross-sectional study showed an inverse association of dietary fiber with NAFLD [50], and this could be explained through the effects of fiber on microbiota as well as on satiation and satiety. UPF, including beverages, usually lead to postprandial hyperglycemia due to a high content of refined carbohydrates, such as white flour and sugar, and simultaneous fiber, water, and protein deprivation [51]. Food glycemic responses have been implicated in liver fat mass accretion through alterations in glucose, insulin, and lipid metabolism [52,53]. In our multivariate analyses, we found that the associations between UPF and liver scores were attenuated but remained statistically significant after further adjustment for these nutritional factors (i.e., saturated and trans FA, fiber, and glycemic load). Mediation analyses revealed that the quality of fat (saturated and trans FA) and carbohydrate (fiber and glycemic load) contributed (11 to 21%) to this pooled effect. This could indicate that these nutritional attributes of UPF might explain part of the observed associations, but clearly not the overall effect. This suggests that mechanisms beyond the nutritional dimension of UPF might also be responsible for the observed associations.

Another potential mechanism responsible for the association between UPF and liver health could be related to additives used during the ultra-processing of these products. In this regard, although the health properties of non-nutritional additives are relatively underexplored in humans, current research performed in rodents and cell lines suggests that they can be harmful for the liver, and the effect could be partly mediated through imbalances of gut microbiota [54]. Some artificial sweeteners (i.e., saccharin, aspartame), emulsifiers (i.e., polysorbate 80), preservatives (i.e., benzoic acid), and flavor enhancers (i.e., monosodium glutamate) could lead to transaminitis, steatosis, degradation, and toxicity in the liver of rodents [54,55,56,57]. We could not explore the potential mediating role of additive content because this information is not yet available in most food composition tables.

In addition, UPF composition may indirectly lead to excessive hepatic lipid accumulation, given their ability to displace healthy foods affecting overall diet quality. In mediation analyses we confirmed that a low adherence to MedDiet explained approximately half of the studied association. Overall diet quality and consumption of UPF are two different but complementary nutritional dimensions to consider in relation to health, given that they could offset one another. A high-quality dietary pattern such as the MedDiet is presumed to be beneficial for NAFLD management [3,4]. An interesting result obtained in our sensitivity analyses suggests that the associations between consumption of UPF and NAFLD indices were attenuated after adjusting for MedDiet adherence, albeit it remained statistically significant. All in all, the potential mechanisms by which UPF consumption may be related to NAFLD is speculative and warrants future studies.

Although not the sole determinant, obesity is recognized as a major risk factor for NAFLD [1,2], whereas type 2 diabetes and MetS are considered as clinical factors that coexist with NAFLD in a bi-directional relationship [58,59]. In this sense, we found that the direct associations between UPF consumption and liver health scores remained significant after adjustment for BMI or waist circumference, albeit their strength markedly diminished. Mediation analyses confirmed that a substantial proportion of the association was driven by obesity, either overall (69%) or abdominal (56% for FLI and 82% for his). In turn, the proportion mediated by type 2 diabetes, MetS, or triglycerides was lower. We have previously reported in the PREDIMED-Plus cohort by using imaging technique that consumption of UPF affected to a similar extent visceral and total fat [12], potentially leading to NAFLD and other diseases. Other large cross-sectional and prospective studies in adults have also shown strong and direct association between UPF and type 2 diabetes [17], MetS [60], and hypertension [42,43]. Regarding markers of liver function, only in the case of the HSI score was the association driven in part by its enzymatic component ALT, and particularly the ALT/AST ratio. The latter has been considered as more accurate than each of these enzymes alone [61]. There is evidence from cross-sectional and cohort studies with healthy adults on a relationship of SSB and fast foods with greater levels of ALT and the ALT/AST ratio [45,62,63]. It needs to be underlined that all PREDIMED-Plus participants were overweight/obese with MetS, and some also had type 2 diabetes (≈27%); hence, future longitudinal studies with healthier individuals are warranted to ascertain what mechanisms underly the association between UPF and liver health.

Beyond the prospective design, control for a wide set of confounders, and performance of a series of sensitivity and stratified analyses, a marked strength of the present study was the use of a large and homogenous sample of men and women within a narrow range of age, BMI, and health conditions. Of note, a unique feature of the present study is that both exposure and outcome were repeatedly measured at the same points in time, potentially decreasing the risk of reverse causality. This is relevant as eating behaviors change over time, given the nutritional intervention given to participants in the PREDIMED-Plus trial [64].

Our study also has limitations. First, its observational nature enabled the identifications of associations only. Second, the participants were older individuals with overweight/obesity and MetS from a Mediterranean area, which limits the generalizability of our findings. However, the described health profile is quite common in modern societies. Third, measurement error is unavoidable when using self-reported dietary data, even though we undertook some actions to improve measurement precision; namely, the FFQ was previously validated in a Spanish population and administrated repeatedly (each 6 months) to the participants by trained dietitians during face-to-face interviews. Moreover, participants with implausible total energy intake values were a priori excluded from analyses, and models were adjusted for total energy intake changes in sensitivity analyses. Fourth, some misclassification in NOVA groups cannot be ruled out, as the FFQ used was not designed to capture details on food processing, and the definition of UPF in the NOVA system is rather broad, allowing multiple interpretations. However, the FFQ items were classified into processing groups with caution and consensus was reached between experts in nutrition and epidemiology. Last but not least, liver fat was estimated based on two surrogate indices, but was not directly measured using imaging techniques. However, both NAFLD algorithms have been validated and have shown good agreement with ultrasonography [37,38].

## 5. Conclusions

In this prospective study we revealed that in older adults with chronic health conditions, consumption of UPF was directly and robustly associated with FLI and HSI scores. Furthermore, these associations were only to a lesser extent explained by the nutritional characteristics of UPF, pointing out the potential and uncovered role of factors related to the processing itself (i.e., non-nutritional chemicals and food matrix breakdown). Future prospective studies in different contexts and with more precise imaging techniques are warranted to confirm our findings on liver fat accumulation, as well as future toxicological, technological, and human experimental studies to clarify underlying mechanisms and develop detection methods for components generated through food processing. With this study we provide novel insights into the recently growing body of evidence on food processing and health risk. The accumulation of firm and high-quality evidence would help global health authorities to update dietary recommendations and food policies by considering criteria of food processing, imposing restrictions to marketing, use of additives, and types of packaging in food technology and trade. Discouragement of UPF consumption and favoring instead fresh or minimally processed foods should be considered by health care providers as a valid preventive and treatment strategy for NAFLD.

## Figures and Tables

**Table 1:** Relative contribution of different food groups to the consumption of UPF in diet of participants at baseline.

Subgroup	Contribution (%)	Item
Sweets	28	chocolate cookies, breakfast cereals, muffins, donuts, croissants, pastries, and confectionery
Non-alcoholic beverages	26	soft drinks (sugar- and artificially-sweetened) and commercial fruit juices
Processed meats	22	ham, chorizo, mortadella, sausages, hamburgers and meat balls, and pate and foie-gras
Pre-prepared dishes, snacks and fast-foods	11	potato chips, croquettes, pizza, instant soups, margarine, mayonnaise, mustard, ketchup, packed fried tomato sauce, and savory packed snacks
Dairy products	11	milkshakes, ice cream, Petit suisse, custard, flan, pudding, and creamy cheese spreads
Alcoholic beverages	2	distillated liquors

Daily intake of beverages was collected in cubic centimeters and then converted into milliliters (1 cc = 1 mL), and further into grams, assuming that 1 mL = 1 g.

**Table 2:** Baseline characteristics of study participants according to baseline sex-specific quintiles of UPF.

		Quintiles of UPF Consumption					
	Total	Q1	Q2	Q3	Q4	Q5	
	Mean (SD)	Mean (SD)	Mean (SD)	Mean (SD)	Mean (SD)	Mean (SD)	*p*-Value
*n*	5867	1174	1173	1173	1173	1173	
**Sociodemographic factors**							
Women, *n* (%)	2807 (47.8)	562 (47.9)	561 (47.8)	562 (47.9)	561 (47.8)	561 (47.8)	
Age (years)	65.0 (4.9)	66.1 (4.7)	65.4 (4.8)	64.8 (4.8)	64.6 (4.9)	64.3 (5.0)	<0.001
Higher education, *n* (%)	1233 (21.0)	234 (19.9)	232 (19.8)	255 (21.7)	254 (21.7)	258 (22.0)	0.087
Current smokers, *n* (%)	732 (12.5)	128 (10.9)	128 (10.9)	146 (12.4)	159 (13.6)	171 (14.6)	0.167
**Lifestyle factors**							
Physical activity (METs min/week)	2477 (2297)	2743 (2481)	2646 (2449)	2439 (2245)	2383 (2160)	2174 (2081)	<0.001
Sedentary behavior (h/day)	6.00 (1.96)	5.68 (1.98)	5.89 (1.91)	6.04 (1.94)	6.18 (1.92)	6.23 (1.97)	<0.001
FFQ:							
Total energy intake (kcal/day)	2360 (550)	2203 (521)	2318 (511)	2371 (536)	2434 (545)	2473 (592)	<0.001
Saturated FA (% of energy intake)	9.95 (1.99)	8.95 (1.80)	9.63 (1.72)	10.1 (1.9)	10.4 (2.0)	10.7 (2.1)	<0.001
Trans FA (% of energy intake)	0.22 (0.13)	0.16 (0.10)	0.20 (0.11)	0.23 (0.12)	0.24 (0.13)	0.27 (0.14)	<0.001
Cholesterol (mg/day)	380 (115)	343 (105)	367 (107)	389 (115)	397 (115)	406 (123)	<0.001
Sodium (mg/day)	3281 (1016)	3021 (983)	3239 (972)	3298 (1010)	3397 (971)	3452 (1088)	<0.001
Glycemic load	131 (46)	123 (45)	128 (42)	131 (45)	133 (47)	138 (49)	<0.001
Fiber intake (g/day)	25.9 (8.7)	27.8 (9.5)	27.0 (8.7)	26.0 (8.3)	25.2 (8.4)	23.6 (7.9)	<0.001
Alcohol intake (g/day)	11.1 (15.1)	12.5 (17.3)	11.6 (15.6)	11.0 (14.3)	10.6 (14.1)	9.70 (14.0)	0.0001
Adherence to erMedDiet (17p score)	8.45 (2.67)	9.61 (2.55)	8.90 (2.67)	8.37 (2.48)	7.98 (2.53)	7.38 (2.55)	<0.001
NOVA food groups:							
Unprocessed or minimally processed foods (% of g/day)	68.1 (12.5)	73.3 (13.4)	71.6 (11.6)	69.7 (10.5)	66.7 (10.3)	59.1 (11.2)	<0.001
Processed culinary ingredients (% of g/day)	2.79 (1.28)	2.69 (1.23)	2.80 (1.28)	2.82 (1.24)	2.88 (1.37)	2.78 (1.26)	0.006
Processed foods (% of g/day)	20.9 (10.8)	21.9 (13.2)	21.4 (11.4)	21.2 (10.1)	21.0 (9.8)	19.0 (8.9)	<0.001
UPF (% of g/day)	8.19 (6.95)	2.12 (0.81)	4.17 (0.63)	6.23 (0.86)	9.44 (1.44)	19.0 (7.9)	<0.001
**Liver health risk factors**							
BMI (kg/m^2^)	32.5 (3.4)	32.0 (3.3)	32.5 (3.4)	32.6 (3.4)	32.7 (3.5)	32.8 (3.6)	<0.001
Overall obesity prevalence, *n* (%)	4289 (73.1)	819 (69.8)	848 (72.3)	870 (74.1)	864 (73.7)	888 (75.7)	0.018
History of overweight from childhood, *n* (%)	334 (5.69)	53 (4.51)	64 (5.46)	78 (6.64)	60 (5.12)	79 (6.73)	0.595
Waist circumference (cm)	107.5 (9.6)	106.3 (9.1)	107.1 (9.3)	107.7 (9.7)	108.2 (9.7)	108.4 (9.9)	<0.001
Abdominal obesity prevalence, *n* (%)	5454 (93.0)	1075 (91.6)	1093 (93.2)	1103 (94.0)	1095 (93.4)	1088 (92.8)	0.224
HbA1c (%)	6.12 (0.87)	6.12 (0.82)	6.14 (0.86)	6.08 (0.82)	6.14 (0.93)	6.11 (0.90)	0.570
Type 2 diabetes prevalence at baseline, *n* (%)	1828 (31.2)	385 (32.8)	363 (31.0)	356 (30.3)	384 (32.7)	340 (29.0)	0.213
Number of MetS factors at baseline	3.38 (0.98)	3.31 (0.99)	3.37 (0.99)	3.40 (0.96)	3.38 (0.98)	3.42 (0.97)	0.069
**Liver health biomarkers**							
FLI (arbitrary units)	77.9 (17.1)	75.8 (17.3)	77.5 (17.4)	78.4 (17.1)	78.7 (16.7)	79.4 (16.8)	<0.001
NAFLD prevalence (FLI ≥ 60), *n* (%)	4934 (84.1)	962 (81.9)	970 (82.7)	990 (84.3)	1000 (85.3)	1012 (86.3)	0.132
HSI (arbitrary units)	43.4 (5.9)	42.7 (4.6)	43.5 (6.5)	43.3 (5.5)	43.4 (4.8)	44.0 (7.4)	<0.001
NAFLD prevalence (HSI ≥ 36), *n* (%)	5585 (95.2)	1117 (95.1)	1124 (95.8)	1119 (95.3)	1115 (95.1)	1110 (94.6)	0.750
ALT (U/L)	27.0 (15.4)	26.4 (15.3)	27.7 (16.2)	26.7 (15.0)	26.5 (14.3)	27.8 (16.0)	0.065
AST (U/L)	23.3 (9.9)	23.1 (9.7)	23.6 (10.4)	23.3 (10.1)	23.2 (9.6)	23.4 (9.8)	0.771
ALT/AST ratio	1.16 (0.53)	1.13 (0.34)	1.18 (0.64)	1.15 (0.46)	1.14 (0.34)	1.21 (0.76)	0.001
AST/ALT ratio	0.95 (0.30)	0.96 (0.28)	0.94 (0.29)	0.96 (0.30)	0.96 (0.30)	0.94 (0.33)	0.204
GGT (U/L)	37.6 (37.2)	37.3 (34.7)	38.5 (40.8)	37.9 (39.7)	37.1 (35.8)	37.1 (34.7)	0.889
Triglycerides (mg/dL)	151 (77)	145 (73)	149 (79)	151 (70)	151 (73)	158 (88)	0.001

Abbreviations: ALT—alanine aminotransferase; AST—aspartate aminotransferase; BMI—body mass index; erMedDiet—energy-restricted Mediterranean Diet; GGT—gamma-glutamyl transferase; FA—fatty acids; FFQ—Food frequency questionnaire; FLI—fatty liver index; HbA1c—glycated hemoglobin; HSI—hepatic steatosis index; MetS—metabolic syndrome; NAFLD—non-alcoholic fatty liver disease; UPF—ultra-processed foods. Sex-specific ranges for quintiles of UPF (%): men—Q1 (lowest): 0.00–3.53, Q2: 3.53–5.44, Q3: 5.45–8.17, Q4: 8.18–12.60, Q5 (highest): 12.62–57.67; women—Q1 (lowest): 0.10–2.97, Q2: 2.97–4.69, Q3: 4.70–6.91, Q4: 6.92–10.64, and Q5 (highest): 10.65–59.48. Values shown are mean (SD) unless otherwise specified. Overall obesity was defined as body mass index ≥ 30.0 kg/m^2^, and abdominal obesity as waist circumference ≥ 88 cm in women or ≥102 cm in men. The consumption of NOVA food groups was expressed as a percentage of total food and beverage intake in g/day. Daily intake of beverages was collected in cubic centimeters and then converted into milliliters (1 cc = 1 mL), and further into grams, assuming that 1 mL = 1 g. *p*-values for comparisons between baseline quintiles of UPF consumption were calculated by one-way ANOVA test for continuous variables and χ2 test for categorical variables.

**Table 3:** Proportion of the association between concurrent changes in UPF consumption (% of g/day, continuous variable) and changes in NAFLD indices during 1 year of follow-up mediated through nutritional factors and NAFLD-related biomarkers (fully adjusted model 2).

(A) FLI Score	
Mediator	% Mediated
**Nutritional factors**	
+changes in total energy intake (kcal/day)	0%
+changes in saturated FA intake (g/day)	19%
+changes in trans FA intake (g/day)	18%
+changes in cholesterol intake (mg/day)	0%
+changes in fiber intake (g/day)	15%
+changes in glycemic load	11%
+changes in sodium intake (mg/day)	0%
+changes in adherence to erMedDiet (17p score)	58%
**NAFLD-related biomarkers**	
+changes in BMI (kg/m^2^)	69%
+changes in waist circumference (cm)	56%
+changes in HbA1c (%)	14%
+changes in number of MetS factors	26%
+changes in GGT (U/L)	0%
+changes in triglycerides (mg/dL)	26%
**(B) HSI Score**	
**Mediator**	**% Mediated**
**Nutritional factors**	
+changes in total energy intake (kcal/day)	0%
+changes in saturated FA intake (g/day)	21%
+changes in trans FA intake (g/day)	17%
+changes in cholesterol intake (mg/day)	0%
+changes in fiber intake (g/day)	0%
+changes in glycemic load	0%
+changes in sodium intake (mg/day)	0%
+changes in adherence to erMedDiet (17p score)	43%
**NAFLD-related biomarkers**	
+changes in BMI (kg/m^2^)	69%
+changes in waist circumference (cm)	82%
+changes in HbA1c (%)	15%
+changes in number of MetS factors	16%
+changes in ALT (U/L)	16%
+changes in AST (U/L)	0%
+changes in ALT/AST	39%

Abbreviations: ALT—alanine aminotransferase; AST—aspartate aminotransferase; BMI—body mass index; erMedDiet—energy-restricted Mediterranean Diet; FA—fatty acids; FLI—Fatty liver index; GGT—gamma-glutamyl transferase; HbA1c—glycated hemoglobin; HSI—Hepatic steatosis index; MetS—metabolic syndrome; NAFLD—non-alcoholic fatty liver disease; UPF—ultra-processed foods. Summary of the mediation analyses was performed to determine the extent to which the association between independent variable (UPF consumption, continuous variable) and each dependent variable (FLI and HSI scores) was mediated through individual nutritional factors (characteristics of UPF and adherence to erMedDiet, as an indicator of healthy dietary pattern) and NAFLD-related biomarkers (known risk factors and components of NAFLD indices). Mediation analyses were performed following standard steps proposed by Baron and Kenny (1986) with adjustments introduced by Iacobucci et al. [39] to evaluate direct and indirect effect and the proportion mediated by each of these variables. More details of this analysis are presented in Supplementary Text S1 and Supplementary Table S5).

## Data Availability

There are restrictions on data availability for the PREDIMED-Plus trial due to the signed consent agreements around data sharing, which only allow access to external researchers for studies following the project purposes. Requestors wishing to access the PREDIMED-Plus trial data used in this study can make a request to the PREDIMED-Plus trial Steering Committee chair: jordi.salas@urv.cat. The request will then be passed to members of the PREDIMED-Plus Steering Committee for deliberation.

## Supplementary Materials

The following supporting information can be downloaded at: https://www.mdpi.com/article/10.3390/nu14194142/s1, Figure S1: Flow chart for the selection of participants for analysis; Figure S2: Directed acyclic graph (DAG); Table S1: Examples of food and beverage items considered as NOVA processing groups; Table S2: Characteristics of the study participants at baseline, 6 months and 12 months of follow-up; Table S3: Association between concurrent changes in UPF consumption (% of g/day) and changes in NAFLD indices during 1-year of follow-up; Table S4: Sensitivity analysis for the association between concurrent changes in UPF consumption (% of g/day) and changes in NAFLD indices during 1-year of follow-up; Table S5: Mediation analysis for the association between concurrent changes in UPF consumption (% of g/day, continuous variable) and changes in NAFLD indices during 1-year of follow-up, through nutritional factors and NAFLD-related biomarkers; Table S6: Association between concurrent changes in UPF consumption (% of g/day) and changes in NAFLD indices during 1-year of follow-up by subgroups; Table S7: Association between concurrent changes in consumption of specific food subgroups within UPF (% of g/day) and changes in NAFLD indices during 1-year of follow-up; Text S1: Procedure for mediation analysis; File S1: Group information.

## Trial Registration

The trial was registered at the International Standard Randomized Controlled Trial (ISRCTN: http://www.isrctn.com/ISRCTN89898870) with number 89898870 and registration date of 24 July 2014, retrospectively registered.

## Abbreviations

ALT	alanine aminotransferase
AST	aspartate aminotransferase
CVD	cardiovascular disease
er	energy-restricted
FA	fatty acids
FFQ	food frequency questionnaire
FLI	fatty liver Index
GGT	gamma-glutamyl transferase
HbA1c	glycated hemoglobin
HDL-c	high-density lipoprotein cholesterol
HSI	hepatic steatosis index
LOCF	last observation carried forward
MedDiet	Mediterranean diet
MetS	metabolic syndrome
NAFLD	non-alcoholic fatty liver disease
PA	physical activity
PREDIMED-Plus	PREvención con DIeta MEDiterránea Plus
SB	sedentary behavior
SSB	sugar-sweetened beverages
TCLSIH	Tianjin Chronic Low-grade Systemic Inflammation and Health Cohort Study
UPF	ultra-processed foods
