# Supplementary material for: Does Consumption of Ultra-Processed Foods Matter for Liver Health? Prospective Analysis among Older Adults with Metabolic Syndrome
Source: Nutrients. 2022 Oct 5;14(19):4142. doi: 10.3390/nu14194142 (PMC9570694; doi:10.3390/nu14194142)
Supplement: Supplementary file 1 [file nutrients-14-04142-s001.zip › nutrients-1931753-supplementary.pdf]

**Does consumption of ultra-processed foods matter for liver health? Prospective analysis among older adults with metabolic syndrome.**

Jadwiga Konieczna, Miguel Fiol, Antoni Colom, Miguel Ángel Martínez-González , Jordi Salas-Salvadó, Dolores Corella, María Trinidad Soria-Florido, J. Alfredo Martínez, Ángel M. Alonso-Gómez, Julia Wärnberg, Jesús Vioque, José López-Miranda, Ramon Estruch, M. Rosa Bernal-López, José Lapetra, Luís Serra-Majem, Aurora Bueno-Cavanillas, Josep A. Tur, Vicente Martín Sánchez, Xavier Pintó, José J. Gaforio, Pilar Matía-Martín, Josep Vidal, Clotilde Vázquez, Lidia Daimiel, Emilio Ros, Maira Bes-Rastrollo, María Pascual, Jose V Sorlí, Albert Goday, María Ángeles Zulet, Anai Moreno-Rodriguez, Francisco Jesús Carmona González, Rafael Valls-Enguix, Juana M Janer, Antonio Garcia-Rios, Rosa Casas, Ana M. Gomez-Perez, José Manuel Santos-Lozano, F. Javier Basterra-Gortari, María Ángeles Martínez, Carolina Ortega-Azorin, Joan Bayó, Itziar Abete, Itziar Salaverria-Lete, Miguel Ruiz-Canela, Nancy Babio, Lourdes Carres, Dora Romaguera.

**Supplementary Figure S1.**

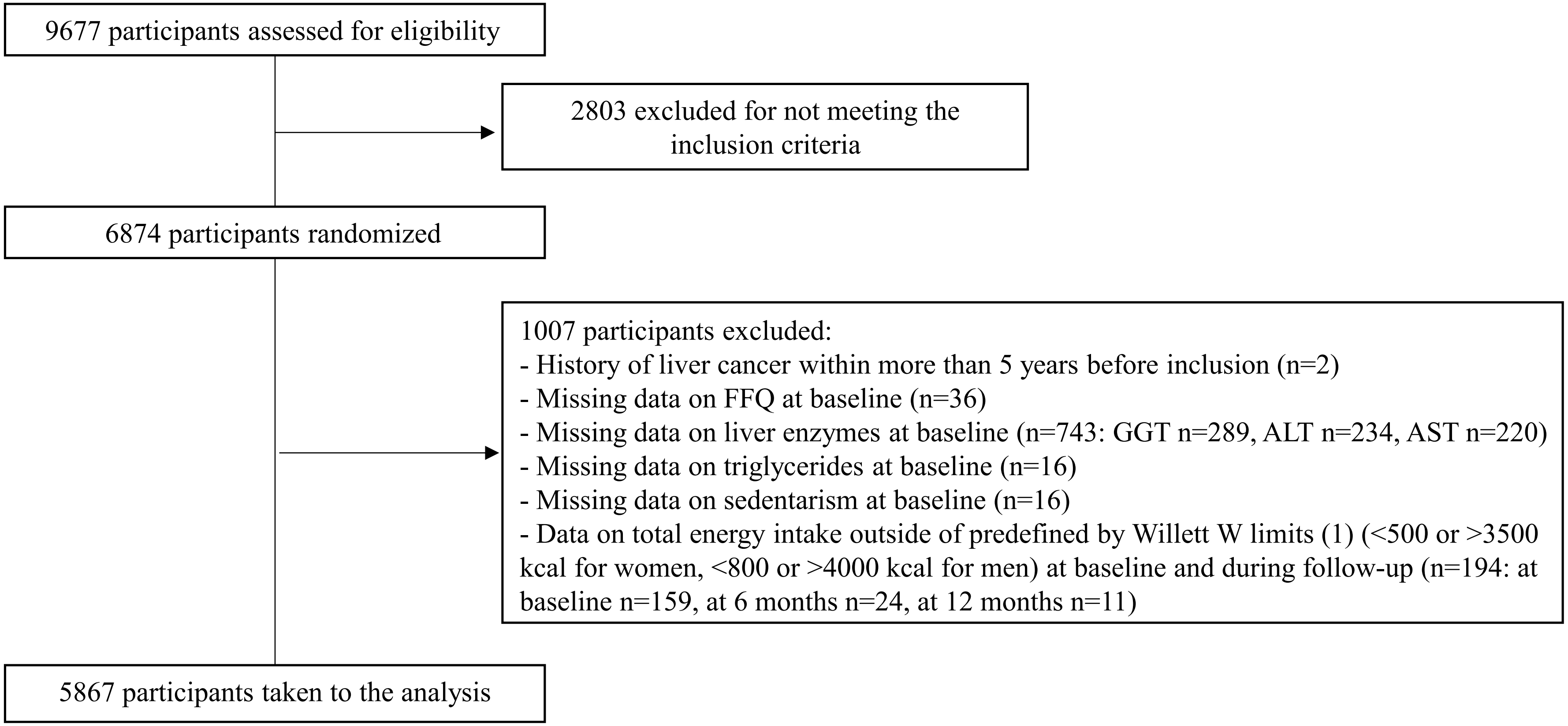


**Flow chart for the selection of participants for analysis.**

Abbreviations: ALT - alanine aminotransferase; AST - aspartate aminotransferase; FFQ – Food frequency questionnaire; FLI – Fatty liver index; GGT - gamma-glutamyltransferase; HSI – Hepatic steatosis index

**Supplementary Table S1. Examples of food and beverage items considered as NOVA processing groups.**

| | |
|---|---|
| Group 1: Unprocessed or minimally processed foods | milk (whole-fat, semi-skimmed and skimmed), yogurt (whole-fat and skimmed), eggs, meats (chicken, turkey, beef, pork, lamb, rabbit), liver, offal, fish and seafoods, fresh vegetables, gazpacho, boiled potatoes, fresh fruits, dried fruits, nuts, legumes, whole-grain cereals, rice (whole-grain and refined), pasta (whole-grain and refined), natural fruit juices, coffee and tea. |
| Group 2: Processed culinary ingredients | vegetable oils (regular and virgin-extra olive oil, oils from sunflower seeds, corn and soybean), butter, lard, salt, sugar and honey |
| Group 3: Processed foods | condensed milk, cream, cheeses (cured, semi-cured, cottage and fresh), bacon, cured ham, canned fish, salt-curing and drying fish, breads (white and whole-grain), artisanal pastries, home-made French fries, olives, fruits in syrup, marmalade, beer, wine, champagne and decaffeinated coffee. |
| Group 4: UPF | Petit suisse, creamy cheese spreads, margarine, custard, flan, pudding, ice-cream, milkshakes, processed meat (ham, chorizo, mortadella, sausages, hamburgers, meat balls, pate, foie-gras), potato chips, breakfast cereals, cookies, industrial and commercial pastries (croissant, ensaimada, donuts, muffins, cakes, churros), chocolates, sugary cocoa powder, marzipan, nougat, pre-prepared dishes (croquettes, empanadillas, pizza), instant soups, mayonnaise, mustard, ketchup, packed fried tomato sauce, savoury packed snacks, soft drinks (sugar- and artificially-sweetened ), commercial fruit juices, alcoholic drinks produced by fermentation followed by distillation (whisky, vodka, gin, liquors) |

**Process of classification of FFQ items into four NOVA groups according to the degree and purpose of their processing.**

The classification of food and beverage items from food frequency questionnaire (FFQ) (2) into one of the four food groups (staring from minimally processed products to ultra-processed foods (UPF)) according to NOVA system (3) was performed by two independent dietitians. Posteriori, the classification was independently revised by specialists in nutritional epidemiology - members of four recruiting centers participating in the trial. Discrepancies in classification were discussed and decision was made by consensus taking some assumptions. For an example, fruit juices, milkshakes, meatballs, hamburgers and pizza can be consumed as artisanal or industrial varieties - we assumed that they were industrial and classified them as ultra-processed products. Regarding yogurts and whole-grain cereals, the FFQ used does not distinguish between plain, sweetened or flavored varieties; these foods were considered to belong to unprocessed or minimally processed foods group.

**Supplementary Text S1. Procedure for mediation analysis.**

Mediation analyses were performed to determine the extent to which the association between independent variable (ultra-processed foods (UPF)), continuous variable) and each dependent variable (Fatty liver index (FLI) and Hepatic steatosis index (HSI)) was mediated through individual nutritional factors, characteristics of UPF (total energy intake, saturated and trans fatty acids, cholesterol, fiber, glycemic index, and sodium), and adherence to energy-restricted Mediterranean Diet, as well as NAFLD-related biomarkers (known risk factors and components of hepatic steatosis indices). Mediation analyses were performed following standard steps proposed by Baron and Kenny (1986) with adjustments introduced by Iacobucci et al (4) to evaluate direct and indirect effect and the proportion mediated by each of these variables, following the schema below.

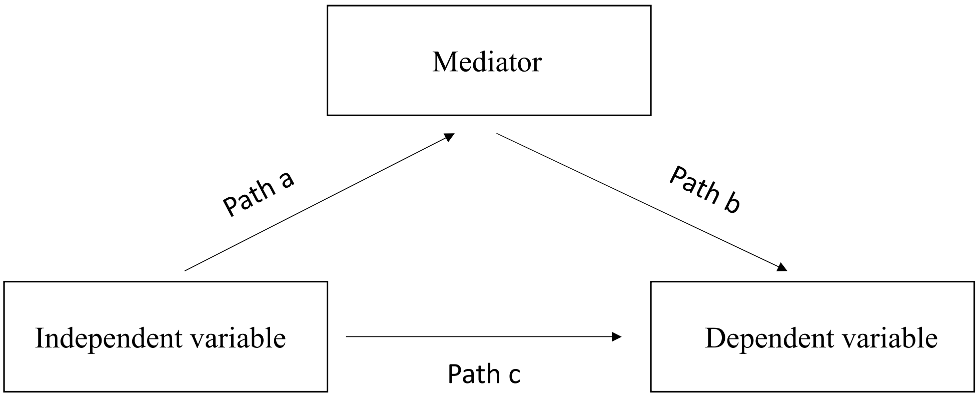


The indirect effect was estimated as the multiplicative product of paths a (the effect of independent variable on the mediator) and b (effect of the mediator on the dependent variable, controlling for the independent variable), whereas the direct effect as the effect of the independent variable on the dependent variable (Path c). Mediation was considered plausible if either Path a or Path b were statistically significant, otherwise the indirect effect and the mediation were considered null. The proportion mediated was calculated as the ratio of indirect effect by the sum of the direct and indirect effect (a*b/(a*b)+c).

For these analyses mixed-effects linear modelling for repeated measure with random intercepts at recruiting center, cluster family and patient level were used after controlling in fully adjusted model 2 for baseline variables, such as age, sex, study arm, educational level, smoking habits, height, as well as repeatedly measured physical activity, sedentary behavior, alcohol intake, and follow-up time.

**Supplementary Table S2. Characteristics of the study participants at baseline, 6 months and 12 months of follow-up.**

| | Baseline | | 6 months | | 12 months | | |
|---|---|---|---|---|---|---|---|
| | **n** | **Mean (SD)** | **n** | **Mean (SD)** | **n** | **Mean (SD)** | ***p-value*** |
| **Sociodemographic factors** | | | | | | | |
| Women, n (%) | 5867 | 2807 (47.8) | | | | | |
| Age (years) | 5867 | 65.0 (4.9) | | | | | |
| Higher education, n (%) | 5867 | 1233 (21.0) | | | | | |
| Current smokers, n (%) | 5867 | 732 (12.5) | | | | | |
| **Lifestyle factors** | | | | | | | |
| Physical activity (METs min/week) | 5867 | 2477 (2297) | 5448 | 2914 (2463) | 5403 | 3042 (2478) | *<0.001* |
| Sedentary behavior (h/day) | 5867 | 6.00 (1.96) | 5449 | 5.98 (1.92) | 5404 | 5.96 (1.89) | *0.011* |
| FFQ: | | | | | | | |
| Total energy intake (kcal/day) | 5867 | 2360 (550) | 5173 | 2213 (450) | 5281 | 2208 (457) | *<0.001* |
| Saturated FA (% of energy intake) | 5867 | 9.95 (1.99) | 5173 | 9.16 (1.72) | 5281 | 9.20 (1.69) | *<0.001* |
| Trans FA (% of energy intake) | 5867 | 0.22 (0.13) | 5173 | 0.15 (0.10) | 5281 | 0.15 (0.10) | *<0.001* |
| Cholesterol (mg/day) | 5867 | 380 (115) | 5173 | 352 (99) | 5281 | 350 (95) | *<0.001* |
| Sodium (mg/day) | 5867 | 3281 (1016) | 5173 | 2906 (873) | 5281 | 2892 (868) | *<0.001* |
| Glycemic load | 5867 | 131 (46) | 5173 | 111 (37) | 5281 | 110 (36) | *<0.001* |
| Fiber intake (g/day) | 5867 | 25.9 (8.7) | 5173 | 29.7 (8.6) | 5281 | 29.6 (8.3) | *<0.001* |
| Alcohol intake (g/day) | 5867 | 11.1 (15.1) | 5173 | 9.62 (13.2) | 5281 | 9.99 (14.0) | *<0.001* |
| Adherence to erMedDiet (17p score) | 5867 | 8.45 (2.7) | 5433 | 11.5 (2.9) | 5386 | 11.7 (2.8) | *<0.001* |
| NOVA processing groups: | | | | | | | |
| Unprocessed or minimally processed foods (% of g/day) | 5867 | 68.1 (12.5) | 5173 | 74.8 (10.8) | 5281 | 74.9 (10.8) | *<0.001* |
| Processed culinary ingredients (% of g/day) | 5867 | 2.79 (1.28) | 5173 | 2.68 (1.07) | 5281 | 2.73 (1.10) | *<0.001* |
| Processed foods (% of g/day) | 5867 | 20.9 (10.8) | 5173 | 17.3 (9.4) | 5281 | 17.4 (9.4) | *<0.001* |
| UPF (% of g/day) | 5867 | 8.19 (6.95) | 5173 | 5.20 (5.29) | 5281 | 5.00 (5.07) | *<0.001* |
| **Liver health risk factors** | | | | | | | |
| BMI (kg/m$^2$) | 5867 | 32.5 (3.4) | 5630 | 31.8 (3.6) | 5628 | 31.7 (3.6) | *<0.001* |
| Overall obesity prevalence, n (%) | 5867 | 4289 (73.1) | 5630 | 3677 (65.3) | 5628 | 3601 (64.0) | |
| History of overweight from childhood, n (%) | 5867 | 334 (5.69) | | | | | |
| Waist circumference (cm) | 5867 | 107.5 (9.6) | 5415 | 105.3 (9.8) | 5365 | 104.8 (10.0) | *<0.001* |

| | | | | | | | |
|---|---|---|---|---|---|---|---|
| Abdominal obesity prevalence, n (%) | 5867 | 5454 (93.0) | 5415 | 4597 (84.9) | 5365 | 4440 (82.8) | |
| HbA1c (%) | 5464 | 6.12 (0.87) | 4817 | 6.06 (0.86) | 4976 | 6.05 (0.82) | *<0.001* |
| Type 2 diabetes prevalence at baseline, n (%) | 5867 | 1828 (31.2) | | | | | |
| Number of MetS factors at baseline | 5844 | 3.38 (0.98) | 5121 | 3.16 (1.06) | 5198 | 3.12 (1.09) | *<0.001* |
| **Liver health biomarkers** | | | | | | | |
| FLI (arbitrary units) | 5867 | 77.9 (17.1) | 5064 | 72.6 (19.8) | 5181 | 71.7 (20.5) | *<0.001* |
| NAFLD prevalence (FLI ≥ 60), n (%) | 5867 | 4934 (84.1) | 5064 | 3768 (74.4) | 5181 | 3761 (72.6) | |
| HSI (arbitrary units) | 5867 | 43.4 (5.87) | 5001 | 42.2 (5.91) | 5097 | 42.0 (5.65) | *<0.001* |
| NAFLD prevalence (HSI ≥ 36), n (%) | 5867 | 5585 (95.2) | 5001 | 4495 (89.9) | 5097 | 4515 (88.6) | |
| ALT (U/L) | 5867 | 27.0 (15.4) | 5184 | 25.1 (17.5) | 5310 | 24.8 (15.6) | *<0.001* |
| AST (U/L) | 5867 | 23.3 (9.92) | 5037 | 22.7 (11.4) | 5140 | 22.9 (16.9) | *0.032* |
| ALT/AST ratio | 5867 | 1.16 (0.53) | 5026 | 1.11 (0.51) | 5125 | 1.09 (0.45) | *<0.001* |
| AST/ALT ratio | 5867 | 0.95 (0.30) | 5026 | 1.00 (0.38) | 5125 | 1.02 (0.75) | *<0.001* |
| GGT (U/L) | 5867 | 37.6 (37.2) | 5135 | 35.4 (36.0) | 5255 | 34.9 (37.1) | *<0.001* |
| Triglycerides (mg/dL) | 5867 | 151 (77) | 5212 | 143 (74) | 5333 | 143 (73) | *<0.001* |

Abbreviations: ALT - alanine aminotransferase; AST - aspartate aminotransferase; BMI – body mass index; erMedDiet – energy-restricted Mediterranean Diet; GGT - gamma-glutamyltransferase; FA – fatty acids; FFQ – Food frequency questionnaire; FLI – fatty liver index; HbA1c – glycated hemoglobin; HSI – hepatic steatosis index; MetS – metabolic syndrome; NAFLD – non-alcoholic fatty liver disease; UPF – ultra-processed foods.

Values shown are mean (SD) unless otherwise specified. Overall obesity was defined as body mass index ≥ 30.0 kg/$m^2$, and abdominal obesity as waist circumference ≥ 88 cm in women or ≥ 102 cm in men.

The consumption of NOVA processing groups was expressed as a percentage of total food and beverage intake in g/day. Daily intake of beverages was collected in cubic centimeters and then converted into milliliters (1 cc = 1 ml), and further into grams, assuming that 1 ml = 1 g.

P-values for changes in repeatedly measured characteristics over follow-up time were determined using mixed-effects linear modelling with random intercepts at recruiting center, cluster family and patient level.

Out of the total analytical sample of 5867 participants, baseline data on HbA1c was available for 93.1% participants and on number of MetS factors for 99.6%. Although all the participants presented MetS (5) at inclusion (factors medically-diagnosed within one year proceeding the inclusion), we recalculated this variable using data on blood parameters and medication use available at baseline and follow-up. Participants with insufficient data on smoking habits were 26 (0.44 %), and were classified in separate category.

**Supplementary Figure S2.**

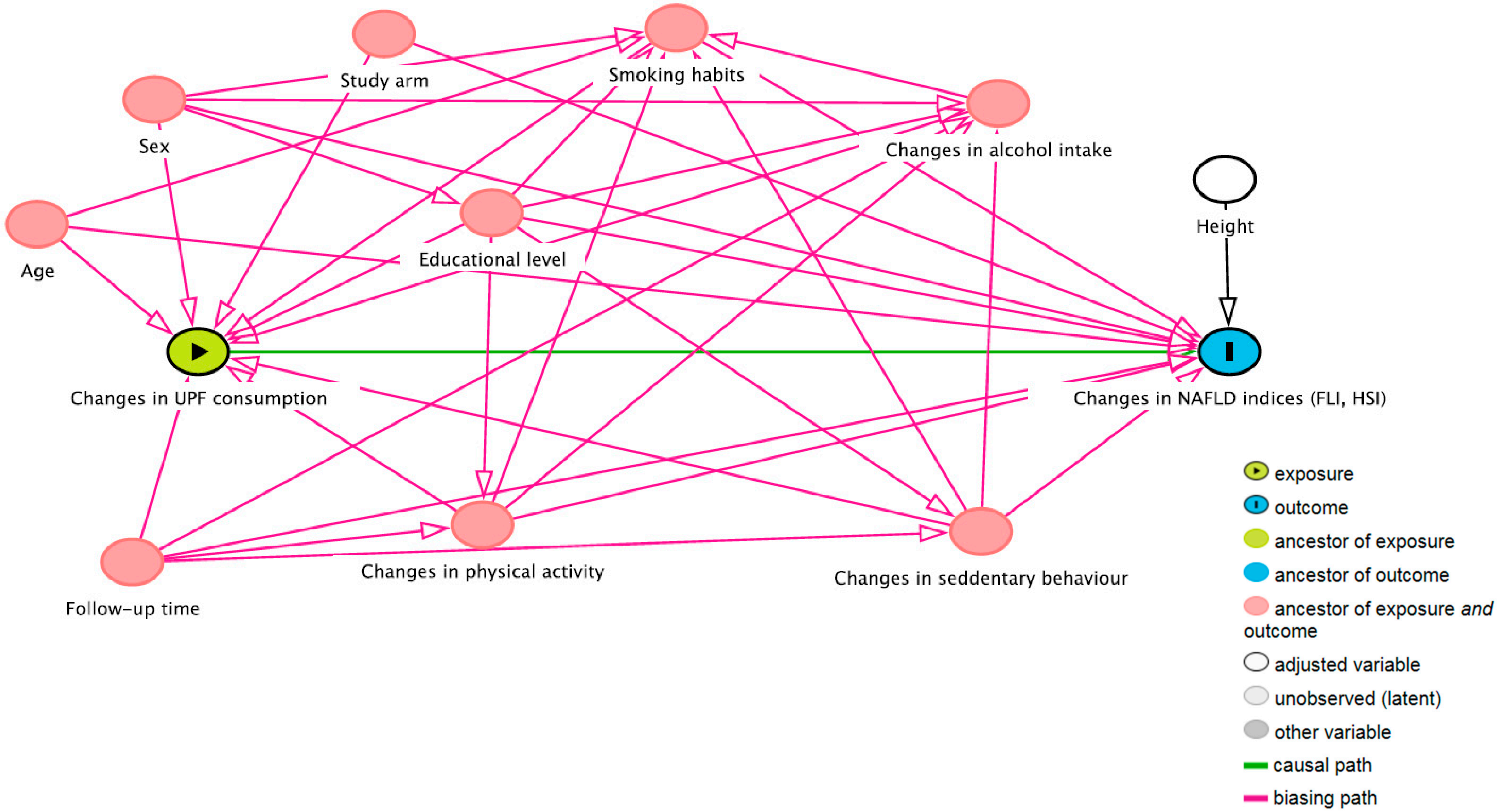

**Figure legend. Directed acyclic graph (DAG).**

The total unconfounded effect of changes in UPF cosnumption on NAFLD indices was drawn and analyzed using available free online application DAGitty (www.dagitty.net). The minimally sufficient adjustment set was age, sex, study arm, baseline educational level, smoking habits, height, as well as changes in physical activity, sedentary behavior, alcohol intake, and follow-up time.

Abbreviations: FLI – fatty liver index; HSI – hepatic steatosis index; NAFLD – non-alcoholic fatty liver disease; UPF – ultra-processed foods.

**Supplementary Table S3. Association between concurrent changes in UPF consumption (% of g/day) and changes in NAFLD indices during 1-year of follow-up.**

| | Continuous[a] | | Quintiles of changes in UPF consumption[b] | | | | | |
|---|---|---|---|---|---|---|---|---|
| | Per 10% change in UPF consumption | | Q1 | Q2 | Q3 | Q4 | Q5 | |
| | **β (95% CI)** | ***p-value*** | **β (95% CI)** | **β (95% CI)** | **β (95% CI)** | **β (95% CI)** | **β (95% CI)** | ***p for trend*** |
| **FLI score** | | | | | | | | |
| Model 1 | 1.88 (1.52; 2.24) | *<0.001* | reference | 0.93 (0.41; 1.46) | 2.31 (1.76; 2.87) | 3.43 (2.85; 4.01) | 4.30 (3.66; 4.93) | *<0.001* |
| Model 2 | 1.60 (1.24; 1.96) | *<0.001* | reference | 0.77 (0.25; 1.28) | 2.01 (1.46; 2.55) | 3.00 (2.43; 3.58) | 3.73 (3.10; 4.35) | *<0.001* |
| **HSI score** | | | | | | | | |
| Model 1 | 0.50 (0.36; 0.64) | *<0.001* | reference | 0.44 (0.22; 0.65) | 0.60 (0.37; 0.83) | 0.70 (0.46; 0.94) | 1.08 (0.83; 1.33) | *<0.001* |
| Model 2 | 0.43 (0.29; 0.57) | *<0.001* | reference | 0.39 (0.17; 0.60) | 0.51 (0.29; 0.74) | 0.58 (0.35; 0.82) | 0.93 (0.67; 1.18) | *<0.001* |

Abbreviations: FLI – Fatty liver index; HSI – Hepatic steatosis index; NAFLD – non-alcoholic fatty liver disease; UPF – ultra-processed foods.

The consumption of UPF was expressed as a percentage of total food and beverage intake in g/day. Daily intake of beverages was collected in cubic centimeters and then converted into milliliters (1 cc = 1 ml), and further into grams, assuming that 1 ml = 1 g.

Mixed-effects linear models for repeated measures with random intercepts at recruiting center, cluster family and patient level were used. Model 1 was adjusted for age, sex, study arm and follow-up time; model 2 was further adjusted for baseline variables, such as educational level, smoking habits, height, as well as repeatedly measured physical activity, sedentary behavior, and alcohol intake.

[a]Estimates β are interpreted as changes in NAFLD indices associated with increments of 10% in UPF. [b]Estimates β are interpreted as changes in NAFLD indices in each sex-specific quintile of UPF consumption, compared to quintile 1, the reference category.

**Supplementary Table S4. Sensitivity analysis for the association between concurrent changes in UPF consumption (% of g/day) and changes in NAFLD indices during 1-year of follow-up.**

| A. FLI score | Continuous[a] | | Quintiles of changes in UPF consumption[b] | | | | | |
|---|---|---|---|---|---|---|---|---|
| | Per 10% change in UPF consumption | | Q1 | Q2 | Q3 | Q4 | Q5 | |
| | β (95% CI) | *p-value* | β (95% CI) | β (95% CI) | β (95% CI) | β (95% CI) | β (95% CI) | *p for trend* |
| Overall | 1.60 (1.24; 1.96) | *<0.001* | reference | 0.77 (0.25; 1.28) | 2.01 (1.46; 2.55) | 3.00 (2.43; 3.58) | 3.73 (3.10; 4.35) | *<0.001* |
| **Nutritional factors** | | | | | | | | |
| + changes in total energy intake (kcal/day) | 1.47 (1.11; 1.84) | *<0.001* | reference | 0.72 (0.20; 1.23) | 1.91 (1.36; 2.46) | 2.86 (2.28; 3.44) | 3.54 (2.90; 4.17) | *<0.001* |
| + changes in saturated FA intake (g/day) | 1.31 (0.94; 1.68) | *<0.001* | reference | 0.64 (0.12; 1.16) | 1.77 (1.21; 2.32) | 2.67 (2.08; 3.26) | 3.29 (2.64; 3.94) | *<0.001* |
| + changes in trans FA intake (g/day) | 1.31 (0.94; 1.68) | *<0.001* | reference | 0.64 (0.12; 1.15) | 1.76 (1.20; 2.31) | 2.66 (2.07; 3.25) | 3.29 (2.65; 3.94) | *<0.001* |
| + changes in cholesterol intake (mg/day) | 1.60 (1.23; 1.96) | *<0.001* | reference | 0.77 (0.26; 1.29) | 2.02 (1.47; 2.57) | 3.02 (2.44; 3.60) | 3.75 (3.12; 4.38) | *<0.001* |
| + changes in fiber intake (g/day) | 1.34 (0.98; 1.70) | *<0.001* | reference | 0.65 (0.14; 1.17) | 1.82 (1.27; 2.37) | 2.71 (2.13; 3.29) | 3.31 (2.68; 3.94) | *<0.001* |
| + changes in glycemic load | 1.43 (1.07; 1.79) | *<0.001* | reference | 0.72 (0.20; 1.23) | 1.89 (1.35; 2.44) | 2.83 (2.25; 3.41) | 3.45 (2.82; 4.08) | *<0.001* |
| + changes in sodium intake (mg/day) | 1.48 (1.12; 1.84) | *<0.001* | reference | 0.71 (0.19; 1.22) | 1.89 (1.34; 2.44) | 2.85 (2.27; 3.43) | 3.52 (2.89; 4.16) | *<0.001* |
| + changes in intake of saturated and trans FA, cholesterol, fiber, glycemic load and sodium | 0.50 (0.12; 0.87) | *0.010* | reference | 0.35 (-0.17; 0.85) | 1.23 (0.67; 1.78) | 1.82 (1.23; 2.42) | 2.02 (1.35; 2.68) | *<0.001* |
| + changes in adherence to erMedDiet (17p score) | 0.68 (0.32; 1.04) | *<0.001* | reference | 0.36 (-0.15; 0.86) | 1.22 (0.68; 1.77) | 1.80 (1.22; 2.38) | 2.08 (1.44; 2.71) | *<0.001* |
| **NAFLD-related risk factors** | | | | | | | | |
| + changes in BMI (kg/m$^2$) | 0.49 (0.21; 0.77) | *<0.001* | reference | -0.01 (-0.42; 0.39) | 0.50 (0.07; 0.93) | 1.02 (0.57; 1.47) | 1.32 (0.83; 1.80) | *<0.001* |
| + changes in waist circumference (cm) | 0.67 (0.40; 0.95) | *<0.001* | reference | 0.35 (-0.06; 0.75) | 0.71 (0.28; 1.14) | 1.27 (0.82; 1.72) | 1.63 (1.14; 2.11) | *<0.001* |
| + changes in HbA1c (%) | 1.25 (0.87; 1.63) | *<0.001* | reference | 0.60 (0.06; 1.14) | 1.84 (1.27; 2.41) | 2.69 (2.08; 3.29) | 3.22 (2.55; 3.88) | *<0.001* |
| + changes in number of MetS factors | 1.22 (0.89; 1.55) | *<0.001* | reference | 0.55 (0.08; 1.02) | 1.57 (1.07; 2.07) | 2.34 (1.81; 2.86) | 2.91 (2.34; 3.49) | *<0.001* |
| + history of overweight | 1.60 (1.24; 1.96) | *<0.001* | reference | 0.76 (0.24; 1.27) | 1.99 (1.45; 2.54) | 2.99 (2.42; 3.57) | 3.72 (3.09; 4.35) | *<0.001* |
| + Type 2 diabetes prevalence at baseline | 1.60 (1.24; 1.96) | *<0.001* | reference | 0.78 (0.26; 1.29) | 2.01 (1.46; 2.56) | 2.99 (2.42; 3.57) | 3.73 (3.10; 4.35) | *<0.001* |
| **Elimination of FLI outliers (1st, 99th percentile)[c]** | 1.53 (1.17; 1.89) | *<0.001* | reference | 0.84 (0.32; 1.36) | 2.06 (1.51; 2.61) | 2.99 (2.42; 3.57) | 3.62 (2.99; 4.24) | *<0.001* |
| **Dealing with missing follow-up data with LOCF** | 1.71 (1.38; 2.04) | *<0.001* | reference | 0.73 (0.25; 1.21) | 1.91 (1.40; 2.42) | 2.86 (2.33; 3.40) | 3.55 (2.97; 4.14) | *<0.001* |

| B. HSI score | Continuous[a] | | Quintiles of changes in UPF consumption[b] | | | | | |
|---|---|---|---|---|---|---|---|---|
| | **Per 10% change in UPF consumption** | | **Q1** | **Q2** | **Q3** | **Q4** | **Q5** | |
| | β (95% CI) | *p-value* | β (95% CI) | β (95% CI) | β (95% CI) | β (95% CI) | β (95% CI) | *p for tren* |
| Overall | 0.43 (0.29; 0.57) | *<0.0001* | reference | 0.39 (0.17; 0.60) | 0.51 (0.29; 0.74) | 0.58 (0.35; 0.82) | 0.93 (0.67; 1.18) | *<0.001* |
| **Nutritional factors** | | | | | | | | |
| + changes in total energy intake (kcal/day) | 0.39 (0.25; 0.54) | *<0.001* | reference | 0.37 (0.16; 0.59) | 0.48 (0.26; 0.71) | 0.54 (0.30; 0.77) | 0.87 (0.61; 1.12) | *<0.001* |
| + changes in saturated FA intake (g/day) | 0.34 (0.20; 0.49) | *<0.001* | reference | 0.34 (0.13; 0.56) | 0.43 (0.20; 0.66) | 0.46 (0.22; 0.70) | 0.77 (0.51; 1.04) | *<0.001* |
| + changes in trans FA intake (g/day) | 0.35 (0.21; 0.50) | *<0.001* | reference | 0.35 (0.14; 0.56) | 0.44 (0.21; 0.67) | 0.48 (0.24; 0.72) | 0.80 (0.54; 1.06) | *<0.001* |
| + changes in cholesterol intake (mg/day) | 0.41 (0.26; 0.55) | *<0.001* | reference | 0.37 (0.16; 0.59) | 0.49 (0.26; 0.71) | 0.54 (0.31; 0.78) | 0.88 (0.63; 1.14) | *<0.001* |
| + changes in fiber intake (g/day) | 0.41 (0.27; 0.55) | *<0.001* | reference | 0.38 (0.17; 0.60) | 0.50 (0.28; 0.73) | 0.56 (0.33; 0.80) | 0.90 (0.65; 1.16) | *<0.001* |
| + changes in glycemic load | 0.39 (0.25; 0.53) | *<0.001* | reference | 0.37 (0.16; 0.59) | 0.49 (0.26; 0.71) | 0.54 (0.31; 0.78) | 0.86 (0.61; 1.12) | *<0.001* |
| + changes in sodium intake (mg/day) | 0.38 (0.24; 0.52) | *<0.001* | reference | 0.36 (0.15; 0.57) | 0.46 (0.23; 0.68) | 0.51 (0.27; 0.75) | 0.83 (0.58; 1.09) | *<0.001* |
| + changes in intake of saturated and trans FA, cholesterol, fiber, glycemic load and sodium | 0.25 (0.10; 0.40) | *0.001* | reference | 0.30 (0.09; 0.52) | 0.35 (0.12; 0.58) | 0.35 (0.11; 0.60) | 0.61 (0.34; 0.88) | *<0.001* |
| + changes in adherence to erMedDiet (17p score) | 0.25 (0.11; 0.40) | *0.001* | reference | 0.30 (0.09; 0.52) | 0.36 (0.13; 0.59) | 0.35 (0.11; 0.59) | 0.60 (0.34; 0.86) | *<0.001* |
| **NAFLD-related risk factors** | | | | | | | | |
| + changes in BMI ($kg/m^2$) | 0.13 (0.01; 0.24) | *0.033* | reference | 0.15 (-0.04; 0.34) | 0.07 (-0.13; 0.27) | 0.04 (-0.17; 0.24) | 0.27 (0.06; 0.48) | *0.015* |
| + changes in waist circumference (cm) | 0.18 (0.06; 0.31) | *0.004* | reference | 0.27 (0.06; 0.47) | 0.18 (-0.03; 0.39) | 0.13 (-0.09; 0.35) | 0.38 (0.15; 0.61) | *0.005* |
| + changes in HbA1c (%) | 0.35 (0.21; 0.50) | *<0.001* | reference | 0.41 (0.19; 0.63) | 0.41 (0.18; 0.65) | 0.48 (0.23; 0.72) | 0.83 (0.57; 1.10) | *<0.001* |
| + changes in number of MetS factors | 0.36 (0.22; 0.49) | *<0.001* | reference | 0.35 (0.14; 0.56) | 0.44 (0.22; 0.67) | 0.46 (0.23; 0.70) | 0.79 (0.54; 1.04) | *<0.001* |
| + history of overweight | 0.43 (0.29; 0.57) | *<0.001* | reference | 0.38 (0.17; 0.60) | 0.51 (0.28; 0.73) | 0.58 (0.34; 0.81) | 0.92 (0.67; 1.17) | *<0.001* |
| + Type 2 diabetes prevalence at baseline | 0.42 (0.28; 0.56) | *<0.001* | reference | 0.40 (0.19; 0.62) | 0.52 (0.30; 0.74) | 0.57 (0.33; 0.80) | 0.92 (0.67; 1.17) | *<0.001* |
| **Elimination of HSI outliers ($1^{st}$, $99^{th}$ percentile)[c]** | 0.32 (0.24; 0.41) | *<0.001* | reference | 0.19 (0.07; 0.31) | 0.34 (0.21; 0.47) | 0.52 (0.39; 0.66) | 0.67 (0.52; 0.82) | *<0.001* |
| **Dealing with missing follow-up data with LOCF** | 0.44 (0.30; 0.57) | *<0.001* | reference | 0.37 (0.17; 0.57) | 0.48 (0.27; 0.69) | 0.57 (0.34; 0.79) | 0.85 (0.61; 1.09) | *<0.001* |

Abbreviations: BMI – body mass index; erMedDiet – energy-restricted Mediterranean Diet; FA – fatty acids; FLI – Fatty liver index; HbA1c – glycated hemoglobin; HSI – Hepatic steatosis index; LOCF – last observation carried forward; MetS – metabolic syndrome; NAFLD – non-alcoholic fatty liver disease; UPF – ultra-processed foods.

The consumption of UPF was expressed as a percentage of total food and beverage intake in g/day. Daily intake of beverages was collected in cubic centimeters and then converted into milliliters (1 cc = 1 ml), and further into grams, assuming that 1 ml = 1 g.

Mixed-effects linear modelling for repeated measures with random intercepts at recruiting center, cluster family and patient level were used after controlling in fully adjusted model 2 for baseline variables, such as age, sex, study arm, educational level, smoking habits, height, as well as repeatedly measured physical activity, sedentary behavior, alcohol intake, follow-up time, and use of antidiabetic medications (for models with HbA1c).

[a]Estimates β are interpreted as changes in NAFLD indices associated with increments of 10% in UPF products consumption. [b]Estimates β are interpreted as changes in NAFLD indices in each sex-specific quintile of UPF consumption, compared to quintile 1, the reference category.

[c]Outliers ($1^{st}$, $99^{th}$ percentile) in the outcome variables were eliminated at baseline and follow-up (for FLI total n=318, for HSI total n=316)

**Supplementary Table S5. Mediation analysis for the association between concurrent changes in UPF consumption (% of g/day, continuous variable) and changes in NAFLD indices during 1-year of follow-up, through nutritional factors and NAFLD-related biomarkers.**

| A. FLI score | Indirect effect | | | | | Direct effect | | % mediated |
|---|---|---|---|---|---|---|---|---|
| | Path a | | Path b | | a*b | Path c | | |
| | β (95% CI) | *p-value* | β (95% CI) | *p-value* | | β (95% CI) | *p-value* | |
| **Nutritional factors** | | | | | | | | |
| + changes in total energy intake (kcal/day) | 124 (112; 136) | *<0.001* | 0.00 (0.00; 0.00) | *<0.001* | 0.00 | 1.47 (1.11; 1.84) | *<0.001* | 0% |
| + changes in saturated FA intake (g/day) | 3.07 (2.88; 3.25) | *<0.001* | 0.10 (0.07; 0.13) | *<0.001* | 0.31 | 1.31 (0.94; 1.68) | *<0.001* | 19% |
| + changes in trans FA intake (g/day) | 0.13 (0.12; 0.14) | *<0.001* | 2.25 (1.62; 2.89) | *<0.001* | 0.29 | 1.31 (0.94; 1.68) | *<0.001* | 18% |
| + changes in cholesterol intake (mg/day) | 19.7 (17.0; 22.5) | *<0.001* | 0.00 (0.00; 0.00) | *0.695* | 0.00 | 1.60 (1.23; 1.96) | *<0.001* | 0% |
| + changes in fiber intake (g/day) | -2.41 (-2.64; -2.19) | *<0.001* | -0.10 (-0.13; -0.08) | *<0.001* | 0.24 | 1.34 (0.98; 1.70) | *<0.001* | 15% |
| + changes in glycemic load | 8.88 (7.83; 9.94) | *<0.001* | 0.02 (0.02; 0.03) | *<0.001* | 0.18 | 1.43 (1.07; 1.79) | *<0.001* | 11% |
| + changes in sodium intake (mg/day) | 197 (174; 221) | *<0.001* | 0.00 (0.00; 0.00) | *<0.001* | 0.00 | 1.48 (1.12; 1.84) | *<0.001* | 0% |
| + changes in adherence to erMedDiet (17p score) | -1.17 (-1.24; -1.10) | *<0.001* | -0.81 (-0.89; -0.74) | *<0.001* | 0.95 | 0.68 (0.32; 1.04) | *<0.001* | 58% |
| **NAFLD-related biomarkers** | | | | | | | | |
| + changes in BMI ($kg/m^2$) | 0.26 (0.22; 0.30) | *<0.001* | 4.26 (4.19; 4.33) | *<0.001* | 1.11 | 0.49 (0.21; 0.77) | *<0.001* | 69% |
| + changes in waist circumference (cm) | 0.54 (0.40; 0.68) | *<0.001* | 1.56 (1.54; 1.59) | *<0.001* | 0.84 | 0.67 (0.40; 0.95) | *<0.001* | 56% |
| + changes in HbA1c (%) | 0.05 (0.04; 0.07) | *<0.001* | 4.13 (3.76; 4.49) | *<0.001* | 0.21 | 1.25 (0.87; 1.63) | *<0.001* | 14% |
| + changes in number of MetS factors | 0.07 (0.04; 0.09) | *<0.001* | 5.93 (5.73; 6.13) | *<0.001* | 0.42 | 1.22 (0.89; 1.55) | *<0.001* | 26% |
| + changes in GGT (U/L) | -0.07 (-0.86; 0.73) | *0.869* | 0.13 (0.13; 0.14) | *<0.001* | 0.00 | 1.61 (1.26; 1.95) | *<0.001* | 0% |
| + changes in triglycerides (mg/dL) | 4.80 (2.90; 6.71) | *<0.001* | 0.09 (0.08; 0.09) | *<0.001* | 0.43 | 1.22 (0.91; 1.54) | *<0.001* | 26% |

| B. HSI score | Indirect effect | | | | | Direct effect | | % mediated |
|---|---|---|---|---|---|---|---|---|
| | Path a | | Path b | | a*b | Path c | | |
| | β (95% CI) | *p-value* | β (95% CI) | *p-value* | | β (95% CI) | *p-value* | β (95% CI) |
| **Nutritional factors** | | | | | | | | |
| + changes in total energy intake (kcal/day) | 124 (112; 136) | *<0.001* | 0.00 (0.00; 0.00) | *0.002* | 0.00 | 0.39 (0.25; 0.54) | *<0.001* | 0% |
| + changes in saturated FA intake (g/day) | 3.07 (2.88; 3.25) | *<0.001* | 0.03 (0.02; 0.04) | *<0.001* | 0.09 | 0.34 (0.20; 0.49) | *<0.001* | 21% |
| + changes in trans FA intake (g/day) | 0.13 (0.12; 0.14) | *<0.001* | 0.57 (0.31; 0.83) | *<0.001* | 0.07 | 0.35 (0.21; 0.50) | *<0.001* | 17% |
| + changes in cholesterol intake (mg/day) | 19.7 (17.0; 22.5) | *<0.001* | 0.00 (0.00; 0.00) | *0.004* | 0.00 | 0.41 (0.26; 0.55) | *<0.001* | 0% |
| + changes in fiber intake (g/day) | -2.41 (-2.64; -2.19) | *<0.001* | -0.01 (-0.02; 0.00) | *0.148* | 0.00 | 0.41 (0.27; 0.55) | *<0.001* | 0% |
| + changes in glycemic load | 8.88 (7.83; 9.94) | *<0.001* | 0.00 (0.00; 0.01) | *<0.001* | 0.00 | 0.39 (0.25; 0.53) | *<0.001* | 0% |
| + changes in sodium intake (mg/day) | 197 (174; 221) | *<0.001* | 0.00 (0.00; 0.00) | *<0.001* | 0.00 | 0.38 (0.24; 0.52) | *<0.001* | 0% |
| + changes in adherence to erMedDiet (17p score) | -1.17 (-1.24; -1.10) | *<0.001* | -0.16 (-0.19; -0.13) | *<0.001* | 0.19 | 0.25 (0.11; 0.40) | *0.001* | 43% |
| **NAFLD-related biomarkers** | | | | | | | | |
| + changes in BMI (kg/m$^2$) | 0.26 (0.22; 0.30) | *<0.001* | 1.12 (1.10; 1.15) | *<0.001* | 0.29 | 0.13 (0.01; 0.24) | *0.033* | 69% |
| + changes in waist circumference (cm) | 0.54 (0.40; 0.68) | *<0.001* | 1.56 (1.54; 1.59) | *<0.001* | 0.84 | 0.18 (0.06; 0.31) | *0.004* | 82% |
| + changes in HbA1c (%) | 0.05 (0.04; 0.07) | *<0.001* | 1.24 (1.10; 1.37) | *<0.001* | 0.06 | 0.35 (0.21; 0.50) | *<0.001* | 15% |
| + changes in number of MetS factors | 0.07 (0.04; 0.09) | *<0.001* | 1.06 (0.98; 1.15) | *<0.001* | 0.07 | 0.36 (0.22; 0.49) | *<0.001* | 16% |
| + changes in ALT (U/L) | 0.59 (0.15; 1.03) | *0.009* | 0.12 (0.11; 0.12) | *<0.001* | 0.07 | 0.36 (0.23; 0.49) | *<0.001* | 16% |
| + changes in AST (U/L) | 0.27 (-0.10; 0.64) | *0.158* | -0.02 (-0.02; -0.01) | *<0.001* | 0.00 | 0.43 (0.29; 0.57) | *<0.001* | 0% |
| + changes in ALT/AST | 0.02 (0.00; 0.03) | *0.009* | 8.21 (8.17; 8.26) | *<0.001* | 0.16 | 0.25 (0.21; 0.29) | *<0.001* | 39% |

Abbreviations: ALT - alanine aminotransferase; AST - aspartate aminotransferase; BMI – body mass index; erMedDiet – energy-restricted Mediterranean Diet; FA – fatty acids; FLI – Fatty liver index; GGT - gamma-glutamyltransferase; HbA1c – glycated hemoglobin; HSI – Hepatic steatosis index; MetS – metabolic syndrome; NAFLD – non-alcoholic fatty liver disease; UPF – ultra-processed foods.

Mediation analyses were performed following procedure described in **Supplementary Text 1**. Briefly, the indirect effect was estimated as the multiplicative product of paths a (the effect of independent variable on the mediator) and b (effect of the mediator on the dependent variable, controlling for the independent variable), whereas the direct effect as the effect of the independent variable on the dependent variable (Path c). Mediation was considered plausible if either Path a or Path b were statistically significant, otherwise the indirect effect and the mediation were considered null. The proportion mediated was calculated as the ratio of indirect effect by the sum of the direct and indirect effect (a*b/(a*b)+c).

Mixed-effects linear modelling for repeated measures with random intercepts at recruiting center, cluster family and patient level were used after controlling in fully adjusted model 2 for baseline variables, such as age, sex, study arm, educational level, smoking habits, height, as well as repeatedly measured physical activity, sedentary behavior, alcohol intake, follow-up time, and use of antidiabetic medications (for models with HbA1c). Estimates β are interpreted as changes in NAFLD indices associated with increments of 10% in UPF consumption.

**Supplementary Table S6. Association between concurrent changes in UPF consumption (% of g/day) and changes in NAFLD indices during 1-year of follow-up by subgroups.**

| Continuous (per 10% change in UPF consumption) | FLI score | | HSI score | |
|---|---|---|---|---|
| | β (95% CI) | *p-value* | β (95% CI) | *p-value* |
| **Sex** | | | | |
| Men (n=3060 (52.2%)) | 1.54 (1.08; 2.00) | *<0.001* | 0.49 (0.30; 0.69) | *<0.001* |
| Women (n=2807 (47.8%)) | 1.66 (1.10; 2.22) | *<0.001* | 0.35 (0.14; 0.56) | *0.001* |
| *p for interaction* | | *0.813* | | *0.208* |
| **Age** | | | | |
| <65 y (n=2688 (45.8%)) | 1.76 (1.25; 2.27) | *<0.001* | 0.52 (0.32; 0.73) | *<0.001* |
| ≥65 y (n=3179 (54.2%)) | 1.50 (1.00; 2.01) | *<0.001* | 0.36 (0.17; 0.56) | *<0.001* |
| *p for interaction* | | *0.639* | | *0.074* |
| **Type 2 diabetes status** | | | | |
| Non-diabetics (n=4039 (68.8%)) | 1.73 (1.29; 2.18) | *<0.001* | 0.42 (0.27; 0.58) | *<0.001* |
| Diabetics (n=1828 (31.2%)) | 1.29 (0.68; 1.90) | *<0.001* | 0.40 (0.10; 0.69) | *0.008* |
| *p for interaction* | | *0.027* | | *0.352* |
| **Alcohol intake** | | | | |
| <20g/day for women and < 30g/d for men (n=5123 (87.3%)) | 1.63 (1.24; 2.01) | *<0.001* | 0.46 (0.31; 0.61) | *<0.001* |
| ≥20g/day for women and ≥ 30g/d for men (n=744 (12.7%)) | 1.52 (0.52; 2.53) | *0.003* | 0.25 (-0.18; 0.67) | *0.253* |
| *p for interaction* | | *0.616* | | *0.908* |
| **Adherence to erMedDiet** | | | | |
| <8 points (n=2171 (37.0%)) | 1.47 (0.97; 1.97) | *<0.001* | 0.41 (0.21; 0.62) | *<0.001* |
| ≥8 points (n=3696 (63.0%)) | 1.66 (1.14; 2.18) | *<0.001* | 0.45 (0.25; 0.65) | *<0.001* |
| *p for interaction* | | *0.830* | | *0.695* |

Abbreviations: erMedDiet – energy-restricted Mediterranean Diet; FLI – Fatty liver index; HSI – Hepatic steatosis index; NAFLD – non-alcoholic fatty liver disease; UPF – ultra-processed foods.

The consumption of UPF was expressed as a percentage of total food and beverage intake in g/day. Daily intake of beverages was collected in cubic centimeters and then converted into milliliters (1 cc = 1 ml), and further into grams, assuming that 1 ml = 1 g.

Mixed-effects linear modelling for repeated measures with random intercepts at recruiting center, cluster family and patient level were used after controlling in fully adjusted model 2 for baseline variables, such as age, sex, study arm, educational level, smoking habits, height, as well as repeatedly measured physical activity, sedentary behavior, alcohol intake, and follow-up time. Estimates β are interpreted as changes in NAFLD indices associated with increments of 10% in UPF consumption.

**Supplementary Table S7. Association between concurrent changes in consumption of specific food subgroups within UPF (% of g/day) and changes in NAFLD indices during 1-year of follow-up.**

| **Continuous (per 10% change in UPF subgroup consumption)** | **FLI score** β (95% CI) | *p-value* | **HSI score** β (95% CI) | *p-value* |
|---|---|---|---|---|
| Dairy products | 2.59 (1.06; 4.13) | *0.001* | 0.36 (-0.27; 0.98) | *0.262* |
| Processed meats | 6.18 (3.88; 8.47) | *<0.001* | 1.75 (0.82; 2.68) | *<0.001* |
| Pre-prepared dishes, snacks and fast-foods | 9.11 (6.14; 12.07) | *<0.001* | 2.17 (0.99; 3.36) | *<0.001* |
| Sweets | 5.32 (3.98; 6.65) | *<0.001* | 1.33 (0.79; 1.87) | *<0.001* |
| Non-alcoholic beverages | 1.03 (0.62; 1.45) | *<0.001* | 0.32 (0.15; 0.48) | *<0.001* |
| Alcoholic beverages | 9.25 (4.62; 13.87) | *<0.001* | 1.22 (-0.60; 3.04) | *0.189* |

Abbreviations: FLI – Fatty liver index; HSI – Hepatic steatosis index; NAFLD – non-alcoholic fatty liver disease; UPF – ultra-processed foods.

The consumption of each specific food group within UPF was expressed as a percentage of total food and beverage intake in g/day. Daily intake of beverages was collected in cubic centimeters and then converted into milliliters (1 cc = 1 ml), and further into grams, assuming that 1 ml = 1 g.

Mixed-effects linear modelling for repeated measures with random intercepts at recruiting center, cluster family and patient level were used after controlling in fully adjusted model 2 for baseline variables, such as age, sex, study arm, educational level, smoking habits, height, as well as repeatedly measured physical activity, sedentary behavior, alcohol intake (except for alcoholic beverages subgroup), and follow-up time. Estimates β are interpreted as changes in NAFLD indices associated with increments of 10% in UPF subgroup consumption.

**File S1: Group information.**

Nonauthor collaborators who contributed to data collection and assistance:

Rovira i Virgili University, Department of Biochemistry and Biotechnology, Human Nutrition Unit, University Hospital of Sant Joan de Reus, Pere Virgili Institute for Health Research, Reus, Spain: R. Pedret Llaberia, R. Gonzalez, R. Sagarra Álamo, F. París Palleja, J. Balsells, J.M. Roca, T. Basora Gallisa, J. Vizcaino, P. Llobet Alpizarte, C. Anguera Perpiñá, M. Llauradó Vernet, C. Caballero, M. Garcia Barco, M.D. Morán Martínez, J. García Rosselló, A. Del Pozo, C. Poblet Calaf, P. Arcelin Zabal, X. Floresví, M. Ciutat Benet, A. Palau Galindo, J.J. Cabré Vila, F. Dolz Andrés, M. Soler, M. Gracia Vidal, J. Vilalta J. Boj Casajuana, M. Ricard, F. Saiz, A. Isach, M. Sanchez Marin Martinez, E. Granado Font, C, Lucena Luque, C. Mestres Sola, N. Becerra-Tomás, J. Basora, G. Mena-Sánchez, L. Barrubés Piñol, N. Rosique-Esteban, S. Chig, I. Abellán Cano, V. Ruiz García, C. Gomez-Martinez, L. Lopez-Gonzalez, A. Salas-Huetos, I. Paz-Graniel, J. Roig Vallverdú, C. Miñana Garcia, L. Sánchez Niembro, P. Hernandez-Alonso, S. Canudas, A. Díaz-López, M. Mendoza Herrera, S. Manzanedo, J. Muralidharan, A. Atzeni, C. Valle. M, Fernández de la Puente, and T. Garcidueñas-Fimbres.

Department of Preventive Medicine and Public Health, University of Navarra-Navarra Institute for Health Research (IdiSNA), Pamplona, Spain: E. Toledo, P. Buil-Cosiales, Z. Vázquez, C. Razquin, A. Gea, A. Sanchez Tainta, B. SanJulian Aranguren, E. Goñi, L. Goñi, M.J. Cobo, A. Rico-Campa, A. Garcia Arellano, J. Diez-Espino, O. Lecea-Juarez, J. Carlos Cenoz-Osinaga, I. Alvarez-Alvarez, M.C. Sayon-Orea, C.I. Fernandez-Lázaro, L. Ruiz-Estigarribia, J. Bartolome-Resano, A. Sola-Larraza (†), E. Lozano-Oloriz, B. Cano-Valles, S. Eguaras, E. Pascual Roquet-Jalmar, I. Galilea-Zabalza, H. Lancova, R. Ramallal, M.L. Garcia-Perez, V. Estremera-Urabayen, M.J. Ariz-Arnedo, C. Hijos-Larraz, C. Fernandez-Alfaro, B. Iñigo-Martinez, R. Villanueva Moreno, S. Martin-Almendros, L. Barandiaran-Bengoetxea, C. Fuertes-Goñi, A. Lezaun-Indurain, M.J. Guruchaga-Arcelus, O. Olmedo-Cruz, L. Escriche-Erviti, R. Ansorena-Ros, R. Sanmatin-Zabaleta, J. Apalategi-Lasa, J. Villanueva-Telleria, M.M. Hernández-Espinosa, L. Herrera-Valdez, L. Dorronsoro-Dorronsoro, Lourdes Echeverria-Lizarraga (†), J.A. Cabeza-Beunza, P. Fernández-Urretavizcaya, P. Gascó-García, C. Royo-Jimenez, J. Moran-Pí, F. Salazar-Fernández, F.J. Chasco-Ros, F. Cortés-Ugalde, J.J. Jurio-Burgui, P. Pascual-Pascual, A.I. Rodríguez-Ezpeleta, M. Esparza-Cáceres, C. Arroyo-Azpa, M. Rodríguez-Sanz de Galdeano, T. Forcen-Alonso, M. Armendariz-Marcotegui, A. Brugos-Larumbe, A. Arillo, and B. López-Aisa.

Department of Preventive Medicine, University of Valencia, University Jaume I, Conselleria de Sanitat de la Generalitat Valenciana, Valencia, Spain: J.I. González, O. Portolés, R. Fernández-Carrión, R. Barragán, E.M. Asensio, O. Coltell, R. Martínez-Lacruz, I. Giménez-Alba, C. Sáiz, R. Osma, E. Férriz, I. González-Monje, P. Guillém-Sáiz, F. Giménez-Fernández, L. Quiles, P. Carrasco, A. Carratalá-Calvo, C. Valero-Barceló, C. Mir, S. Sánchez-Navarro, J. Navas, I. González-Gallego, L. Bort-Llorca, L. Pérez-Ollero, M. Giner-Valero, R. Monfort-Sáez, J. Nadal-Sayol, V. Pascual-Fuster, M. Martínez-Pérez, C. Riera, M.V. Belda, A. Medina, E. Miralles, M.J. Ramírez-Esplugues, M. Rojo-Furió, G. Mattingley, M.A. Delgado, M.A. Pages, Y Riofrío, L. Abuomar, N. Blasco-Lafarga, R. Tosca, L. Lizán, A.M Valcarce, M.D. Medina, S. de Valcárcel, N. Tormo, O. Felipe-Román, S. Lafuente, E.I. Navío, G. Aldana, J.V. Crespo, J.L. Llosa, L. González-García, and J.J. Tamarit.

Cardiovascular Risk and Nutrition Research Group, Endocrinology Service, Neurosciences Programme, Clinical Research Unit at the Hospital del Mar Medical Research Institute (IMIM), Barcelona. Medicine Departament, Universitat Autònoma de Barcelona, Barcelona, Spain: M.

Fitó, O. Castañer, M.A. Muñoz, M.D. Zomeño, A. Hernaéz, L. Torres, M. Quifer, R. Llimona, G Freixer, KA. Pérez-Vega, M. Farràs, R. Elosua, J. Vila, I. Subirana, S. Pérez, J.J. Chillaron Jordan, J.A. Flores Lerroux, D. Benaiges Boix, G. Llauradó, M. Farré, E. Menoyo, A. Aldea-Perona, M. Pérez-Otero, D. Muñoz-Aguayo, S. Gaixas, G. Blanchart, A. Sanllorente, J. Valussi, A. Cuenca, L. Forcano, A. Pastor, A. Boronat, S. Tello, M. Cabañero, L. Franco, H. Schröder, R. De la Torre, C. Medrano, M.T. García, V. Robledo, P. Babi, E. Canals, N. Soldevila, C. Roca, M.S. Comas, G. Gasulla, X. Herraiz, A. Martínez, E. Vinyoles, J.M. Verdú, M. Masague Aguade, E. Baltasar Massip, M. López Grau, M. Mengual, V. Moldon, M. Vila Vergaz, R. Cabanes Gómez, Ciurana, M. Gili Riu, A. Palomeras Vidal, F Peñas F, A Raya, M.A. Sebastian, M. Valls, J. Guerrero, M. Marne, E. Minguella, M. Montenegro, A. Sala, M.R. Senan, N. Talens, and N. Vera.

Nutritional Epidemiology Unit, Miguel Hernandez University, ISABIAL-FISABIO, Alicante, Spain: M. García-de-la-Hera, S. Gonzalez-Palacios, L. Torres-Collado, L. Compañ-Gabucio, A. Oncina-Canovas, L. Notario-Barandiaran, D. Orozco-Beltran, S. Pertusa Martínez, A. Asencio, I. Candela-García, J.M. Zazo, D. Vivancos Aparicio, N. Fernández-Brufal, J. Román Maciá, F. Ortiz Díaz, M. García Muñoz, C. Barceló, E. Martínez-García, M Damaj-Hamieh, M.C. Martínez Vergara, M.A. Sempere Pascual, S.J. Miralles Gisbert, A. González Botella, C.M. López García, N. Gómez Bellvert, V. Martínez Avilés, R. Lloret Macián, A. Pastor Morel, M. Mayor-Llorca, J.J. Ballester Baixauli, G. Notario García, M.A. Belmar-Bueno, E.P. Cases Pérez, C. Tercero Maciá, L.A. Mira Castejón, J. Torregrosa García, C. Pastor Polo, E. Puig Agulló, M.V. Hernándis Marsán, M.J. González Fajardo, I. Hervella Durantez, M.C. Latorre Use, A. Bernabé Casanova, F. Medina Ruzafa, E. Robledano, I. Vilanova Martínez, and A. Molina Santiago.

Hospital Son Espases (HUSE) and Institute for Health Research Illes Balears (IdISBa), Palma de Mallorca, Spain: M. Moñino, M. Morey, A.M. Galmés-Panadés, M.A. Martín, E. Rayó, J. Llobera, J. Fernández-Palomeque, E. Fortuny, M. Noris, L. López, X. Rosselló, S. Munuera, F. Tomás, F. Fiol, A. Jover, C. Vallespir, I. Mattei, N. Feuerbach, M. del Mar Sureda, S. Vega, L. Quintana, A. Fiol, M. Amador, S. González, J. Coll, A. Moyá, T. Piqué Sistac, M.D. Sanmartín Fernández, M.C. Piña Valls, M.A. Llorente San Martín, and J. Pou Bordoy.

Department of Nutrition: Food Sciences, and Physiology, Center for Nutrition Research, University of Navarra, Pamplona, Spain: I. Cantero, C. Cristobo, I. Ibero-Baraibar, J. Ágreda-Peiró, M.D. Lezáun-Burgui, N. Goñi-Ruiz, R. Bartolomé-Resano, E. Cano-Cáceres, T. Elcarte-López, E. Echarte-Osacain, B. Pérez-Sanz, I. Blanco-Platero, A. Andueza- Azcárate, A. Gimeno-Aznar, E. Ursúa-Sesma, B. Ojeda-Bilbao, J. Martinez-Jarauta, L. Ugalde-Sarasa, B. Rípodas-Echarte, M.V. Güeto-Rubio, C. Napal-Lecumberri, MD Martínez-Mazo, E Arina-Vergara, A. Parra-Osés, F. Artal-Moneva, F. Bárcena-Amigo, F. Calle-Irastoza, J. Abad-Vicente, J.I. Armendáriz-Artola, P. Iñigo-Cibrian, J. Escribano-Jarauta, J. Ulibarri-delportillo, B. Churio-Beraza, Y. Monzón-Martínez, E. Madoz-Zubillaga, and C. Arroniz.

University of Málaga and Institute of Biomedical Research in Malaga (IBIMA), Málaga, Spain: F.J. Barón-López, J.C. Fernández García, N. Pérez-Farinós, N. Moreno-Morales, M. del C. Rodríguez-Martínez, J. Pérez-López, J.C. Benavente-Marín, E. Crespo Oliva, E. Contreras Fernández, R. Carabaño Moral, S. Torres Moreno, M.V. Martín Ruíz, M. Alcalá Cornide, and V. Fuentes Gómez.

Lipids and Atherosclerosis Unit, Department of Internal Medicine, Maimonides Biomedical Research Institute of Cordoba (IMIBIC), Reina Sofia University Hospital, University of Cordoba, Cordoba, Spain: J. Criado García, A.I. Jiménez Morales, A. Ortiz Morales, J.D. Torres Peña,

F.J. Gómez Delgado, J.F. Alcalá, A. León Acuña, A.P. Arenas Larriva, F. Rodríguez Cantalejo, J. Caballero Villaraso, I. Nieto Eugenio, P. Coronado Carvajal, M.C del Campo Molina, P.J. Peña Orihuela, I. Perez Corral, and G. Quintana Navarro.

Department of Internal Medicine, Institut d'Investigacions Biomèdiques August Pi i Sunyer (IDIBAPS), Hospital Clínic, University of Barcelona, Barcelona, Spain: M. Domenech, C. Viñas, S. Castro-Barquero, A.M. Ruiz-León, R. Losno, L. Tarés, A. Jordán, R. Soriano, M. Camafort, C. Sierra, E. Sacanella, A. Sala-Vila, J. M. Cots, I. Sarroca, M. García, N. Bermúdez, A. Pérez, I. Duaso, A. de la Arada, R. Hernández, C. Simón, M.A. de la Poza, I. Gil, M. Vila, C. Iglesias, N. Assens, M. Amatller, LL. Rams, T. Benet, G. Fernández, J. Teruel, A. Azorin, M. Cubells, D. López, J.M. Llovet, M.L. Gómez, P. Climente, L. de Paula, J. Soto, C. Carbonell, C. Llor, X. Abat, A. Cama, M. Fortuny, C. Domingo, A. I. Liberal, T. Martínez, E. Yañez, M. J. Nieto, A. Pérez, E. Lloret, C. Carrazoni, A. M. Belles, C. Olmos, M. Ramentol, M. J. Capell, R. Casas, I. Giner, A. Muñoz, R. Martín, E. Moron, A. Bonillo, G. Sánchez, C. Calbó, J. Pous, M. Massip, Y. García, M.C. Massagué, R. Ibañez, J. Llaona, T. Vidal, N. Vizcay, E. Segura, C. Galindo, M. Moreno, M. Caubet, J. Altirriba, G. Fluxà, P. Toribio, E. Torrent, J. J. Anton, A. Viaplana, G. Vieytes, N. Duch, A. Pereira, M. A. Moreno, A. Pérez, E. Sant, J. Gené, H. Calvillo, F. Pont, M. Puig, M. Casasayas, A. Garrich, E. Senar, A. Martínez, I. Boix, E. Sequeira, V. Aragunde, S. Riera, M. Salgado, M. Fuentes, E. Martín, A. Ubieto, F. Pallarés, C. Sala, A. Abilla, S. Moreno, E. Mayor, T. Colom, A. Gaspar, A. Gómez, L. Palacios, and R. Garrigosa.

Departament of Preventive Medicine and Public Health, University of Granada, Granada, Spain: L. García Molina, B. Riquelme Gallego, N. Cano Ibañez, A. Maldonado Calvo, A. López Maldonado, E.M. Garrido, A. Baena Dominguez, F. García Jiménez, E. Thomas Carazo, A. Jesús Turnes González, F. González Jiménez, F. Padilla Ruiz, J. Machado Santiago, M.D. Martínez Bellón, A. Pueyos Sánchez, L. Arribas Mir, R. Rodríguez Tapioles, F. Dorador Atienza, L. Baena Camus, C. Osorio Martos, D. Rueda Lozano, M. López Alcázar, F. Ramos Díaz, M. Cruz Rosales Sierra, P. Alguacil Cubero, A. López Rodriguez, F. Guerrero García, J. Tormo Molina, and F. Ruiz Rodríguez.

Bioaraba Health Research Institute, Cardiovascular, Respiratory and Metabolic Area; Osakidetza Basque Health Service, Araba University Hospital; and University of the Basque Country UPV/EHU, Vitoria-Gasteiz, Spain: M.C. Belló, L. Tojal, L. Goicolea, C. Sorto, A Goikoetxea, A. Casi Casanellas, M.L. Arnal Otero, J. Ortueta Martínez De Arbulo, J. Vinagre Morgado, J. Romeo Ollora, J. Urraca, M.I. Sarriegui Carrera, F.J. Toribio, E. Magán, A. Rodríguez, S. Castro Madrid, M.T. Gómez Merino, M. Rodríguez Jiménez, M. Gutiérrez Jodra, B. López Alonso, J. Iturralde Iriso, C. Pascual Romero, and A. Izquierdo De La Guerra.

Research Group on Community Nutrition & Oxidative Stress, University of Balearic Islands, Palma de Mallorca, Spain: M. Abbate, E. Angullo, E. Argelich, M.M. Bibiloni, C. Bouzas, X. Capó, S. Carreres, L. Gallardo, J.M. Gámez, B. García, C. García, A. Julibert, C. Gómez, I. Llompart, A. Martorell, C.M. Mascaró, D. Mateos, M, Monserrat, S. Montemayor, A. Pons, A. Pouso, J. Ramos, V. Ramos, T. Ripoll, T. Rodríguez, L. Sanz, A. Sureda, S. Tejada, and L. Ugarriza.

Virgen de la Victoria Hospital, University of Málaga, Málaga, Spain: M. Macías González, J. Ruiz Nava, J.C. Fernández García, A. Muñoz Garach, A. Vilches Pérez, A. González Banderas, A.V. Alarcón-Martín, M. García Ruiz de Mier, J. Alcaide Torres, A. Vargas Candela, M. León Fernández, R. Hernández Robles, S. Santamaría Fernández, and J.M. Marín.

University of Las Palmas de Gran Canaria, Las Palmas, Spain: J. Álvarez-Pérez, E.M. Díaz Benítez, F. Díaz-Collado, A. Sánchez-Villegas, J. Pérez-Cabrera, L.T. Casañas-Quintana, R.B. García-Guerra, I. Bautista-Castaño, C. Ruano-Rodríguez, F. Sarmiento de la Fe, J.A. García-Pastor, B. Macías-Gutiérrez, I. Falcón-Sanabria, C. Simón-García, A.J. Santana-Santana, J.B. Álvarez-Álvarez, B.V. Díaz-González, J.M. Castillo Anzalas, R.E. Sosa-Also, and J. Medina-Ponce.

Biomedicine Institute (IBIOMED), University of León and Primary Health Care Management of León (Sacyl), León, Spain: Biomedicine Institute (IBIOMED); University of León, and Primary Health Care Management of León (Sacyl), León, Spain: S. Abajo Olea, L. Álvarez-Álvarez, M. Rubín García, A. Torres, P. Farias, N. Cubelos, A. Adlbi Sibai, M. Ajenjo, E. Carriedo Ule, M. Escobar Fernández, J.I. Ferradal García, J.P. Fernández Vázquez, C. González Quintana, F. González Rivero, M. Lavinia Popescu, J.I. López Gil, J. López de la Iglesia, A. Marcos Delgado, C. Merino Acevedo, S. Reguero Celada, M. Rodríguez Bul, and E. Fernández Mielgo.

Department of Family Medicine, Distrito Sanitario Atención Primaria Sevilla, Sevilla, Spain: L. Miró-Moriano, C. Domínguez-Espinaco, S. Vaquero-Díaz, F.J. García-Corte, A. Santos-Calonge, C. Toro-Cortés, N. Pelegrina-López, V. Urbano-Fernández, M. Ortega-Calvo, J. Lozano-Rodríguez, I. Rivera-Benítez, M. Caballero-Valderrama, P. Iglesias-Bonilla, P. Román-Torres, Y. Corchado-Albalat, and L. Mellado-Martín.

**Department of Endocrinology and Nutrition, Hospital Fundación Jimenez Díaz. Instituto de Investigaciones Biomédicas IISFJD. University Autonoma, Madrid, Spain:** A.I. de Cos, S. Gutierrez, S. Artola, A. Galdon, I. Gonzalo.

Lipids and Vascular Risk Unit, Internal Medicine, University Hospital of Bellvitge (IDIBELL) and Hospitalet de Llobregat, Barcelona, Spain: A. Galera, M. Gimenez-Gracia, E. de la Cruz, R. Figueras, M. Poch, R. Freixedas, F. Trias, I. Sarasa, M. Fanlo-Maresma, H. Lafuente, M. Liceran, A. Rodriguez-Sanchez, C. Pallarols, E. Gómez-Sanchez, V. Esteve-Luque, J. Monedero, X. Corbella, and E. Corbella.

Department of Endocrinology, IDIBAPS, Hospital Clinic, University of Barcelona, Barcelona, Spain: A. Altés, I. Vinagre, C. Mestre, J. Viaplana, M. Serra, J. Vera, T. Freitas, E. Ortega, I. Pla, and R. Olbeyra.

Nutritional Control of the Epigenome Group, Precision Nutrition and Obesity Program, Institute IMDEA-Food, CEI UAM+CSIC, Madrid, Spain: J.M. Ordovás, V. Micó, L. Berninches, L. Díez, M.J. Concejo, J. Muñoz, M. Adrián, Y. de la Fuente, C. Albertos, M.L. Cornejo, C. Cuesta. A. Montero., J. Aroca, B. Cáceres Sánchez, ME. Jiménez Caravera, MA. Aldavero Palacios, S. Conti Fernández, MC. Rodríguez Romero, PJ. Jiménez Pérez, V. Fernández Gutiérrez, Y. De La Fuente Cortes, R. Baños Morras, C. Gómez Almodóvar, P. González Escobar, AM. Ibarra Sánchez, A. Manzanares Briega M. Renata Muñoz Bieber, J. Muñoz Gutiérrez, L. Santos Larregola, C. Cassinello Espinosa, MC. Molins Santos, JM. Rodríguez Buitrago, E. Sánchez Balsalobre, C. Albertos Carrion, C. Cuesta González, L. González Torres, ME. Villahoz Loureiro, E. Arrebola Vivas, AF. Fernández Garcia, ML. Cornejo Alonso, JA. Romo Martin, L. Carabias Jaen, C. Barbero Macías, MA. Blanca De Miguel Oteo, E. Bartolomé Cobeña, MM. Adrián Sanz, MA. Angel Álvaro Sánchez, MD. Cano Pérez, MP. Lopez Morandeira, A. Montero Costa, E. Robles Fernandez, I. Alba Llacer, J. Aroca Palencia, R. Sanz Merino, MJ. Concejo Carranza, A. Garcia Romero, MC. Gómez Tabera, C. Lesmes Lora, J. Zarco Montejo, A. Campo Lopez, ME. Collado Correa, MS. Díaz Moreno, B. Doval Segura, RM. Gómez Quiroga, S. Hernando Gómez, MJ. Martínez Sanz, and AM. Yunquera Alonso

Division of Preventive Medicine, University of Jaén, Jaén, Spain: S. Moraleda, N. Liétor, J.I. Peis, T. Ureña, M. Rueda, and M.I. Ballesta.

Department of Endocrinology and Nutrition, Hospital Clínico San Carlos, Instituto de Investigación Sanitaria del Hospital Clínico San Carlos (IdISSC), Madrid, España (Spain para internacionales: C. Moreno Lopera, C. Aragoneses Isabel, M.A. Sirur Flores, M. Ceballos de Diego, T. Bescos Cáceres, Y. Peña Cereceda, M. Martínez Abad, R. Cabrera Vélez, M. González Cerrajero, M.A. Rubio Herrera, M. Torrego Ellacuría, A. Barabash Bustelo, M. Ortiz Ramos, and A. Larrad Sainz.

Support groups: C. Botella, F. Fernandez-Aranda, R. Lamuela, A. Marcos, M.P. Portillo, E. Ros, G. Sáez, F. Arós, and E. Gómez-Gracia.

## References

1. Willett W. Nutritional epidemiology. 3rd ed. Oxford University Press; 2013. p. 529.

2. Fernández-Ballart JD, Piñol JL, Zazpe I, Corella D, Carrasco P, Toledo E, et al. Relative validity of a semi-quantitative food-frequency questionnaire in an elderly Mediterranean population of Spain. Br J Nutr. 2010;103(12):1808–16.

3. Monteiro CA, Cannon G, Levy RB, Moubarac J-C, Louzada ML, Rauber F, et al. Ultra-processed foods: what they are and how to identify them. Public Health Nutr. 2019 Apr 12;22(5):936–41.

4. Iacobucci D, Saldanha N, Deng X. A Meditation on Mediation: Evidence That Structural Equations Models Perform Better Than Regressions. J Consum Psychol. 2007 Apr 1;17(2):139–53.

5. Alberti KGMM, Eckel RH, Grundy SM, Zimmet PZ, Cleeman JI, Donato KA, et al. Harmonizing the Metabolic Syndrome: A Joint Interim Statement of the International Diabetes Federation Task Force on Epidemiology and Prevention; National Heart, Lung, and Blood Institute; American Heart Association; World Heart Federation; International. Circulation. 2009;120(16):1640–5.
